# Pluripotent stem cell-derived committed cardiac progenitors remuscularize damaged ischemic hearts and improve their function in pigs

**DOI:** 10.1038/s41536-023-00302-6

**Published:** 2023-05-26

**Authors:** Lynn Yap, Li Yen Chong, Clarissa Tan, Swarnaseetha Adusumalli, Millie Seow, Jing Guo, Zuhua Cai, Sze Jie Loo, Eric Lim, Ru San Tan, Elina Grishina, Poh Loong Soong, Narayan Lath, Lei Ye, Enrico Petretto, Karl Tryggvason

**Affiliations:** 1 Cardiovascular & Metabolic Disorders Program, Duke-NUS Medical School, National University of Singapore, Singapore, 169857 Singapore; 2 Lee Kong Chian School of Medicine, Nanyang Technological University, 11 Mandalay Road, Singapore, 308232 Singapore; 3 National Heart Research Institute Singapore, National Heart Centre Singapore, Singapore, 169609 Singapore; 4 Ternion Biosciences, Singapore, 574329 Singapore; 5 Cardiovascular Disease Translational Research Program, Yong Loo Lin School of Medicine, NUS, Singapore, 169609 Singapore; 6 Department of Biomedical Engineering, University of Alabama, Birmingham, 35233 England; 7 Department of Medicine Duke University, Durham, NC 27710 USA; 8 Department of Medical Biochemistry and Biophysics, Karolinska Institute, 171 77 Stockholm, Sweden

**Keywords:** Regenerative medicine, Embryonic stem cells

## Abstract

Ischemic heart disease, which is often associated with irreversibly damaged heart muscle, is a major global health burden. Here, we report the potential of stem cell-derived committed cardiac progenitors (CCPs) have in regenerative cardiology. Human pluripotent embryonic stem cells were differentiated to CCPs on a laminin 521 + 221 matrix, characterized with bulk and single-cell RNA sequencing, and transplanted into infarcted pig hearts. CCPs differentiated for eleven days expressed a set of genes showing higher expression than cells differentiated for seven days. Functional heart studies revealed significant improvement in left ventricular ejection fraction at four and twelve weeks following transplantation. We also observed significant improvements in ventricular wall thickness and a reduction in infarction size after CCP transplantation (*p*-value < 0.05). Immunohistology analyses revealed in vivo maturation of the CCPs into cardiomyocytes (CM). We observed temporary episodes of ventricular tachyarrhythmia (VT) in four pigs and persistent VT in one pig, but the remaining five pigs exhibited normal sinus rhythm. Importantly, all pigs survived without the formation of any tumors or VT-related abnormalities. We conclude that pluripotent stem cell-derived CCPs constitute a promising possibility for myocardial infarction treatment and that they may positively impact regenerative cardiology.

## Introduction

Ischemic heart disease is the most common cause of human death worldwide^1^. For over two decades, cell therapy approaches, including stem cell-based methods, have been considered a possible solution for myocardial infarction (MI), a clinical condition of ischemic heart disease, but attempts to develop such treatments have thus far been unsuccessful^2–4^. This includes earlier studies on transplantation of human pluripotent stem cells (hPSCs)-derived immature cardiomyocytes (CMs) into experimentally infarcted pigs and nonhuman primates (NHP)^5,6^. While successful engraftment has been observed in infarcted host hearts, severe side effects in the form of graft-induced tachyarrhythmia and subsequent death predominantly have hindered their use in human patients. Although results of one study have suggested that pharmacological means or overdrive pacing of transplanted CMs cells can circumvent the temporary occurrence of tachycardia, such immature CMs have not yet proved fully effective in cardiac regeneration^7^. Other studies have reported that mature hPSC-derived ventricular CMs can form more mature grafts in infarcted rat hearts^8^ and guinea pig models^9^, as compared to immature CMs. However, such small animal models are not suitable for identifying the incidence of ventricular arrhythmia, and therefore, the clinical potential of mature CMs remains unknown. Yet another study has shown that early ventricular progenitor cells expressing *ISL1* can preserve heart function in infarcted murine hearts^10^, suggesting the applicability of these cells over CMs in cell therapy. However, since these progenitors were differentiated on undefined mouse tumor extract (Matrigel®), and were found unsuitable for clinical use. Moreover, the authors did not investigate the incidence of graft-induced arrhythmia which is an important limitation of cellular therapy.

The cell type that is possibly most suitable for clinical use was identified by Menasché’s group, in phase 1 clinical trial (ESCORT) in severe heart failure patients^11,12^. They successfully placed a fibrin patch transplanted with human embryonic stem cells (hESCs)-derived *CD15*+ and *ISL1*+ progenitors into the epicardium of the infarcted hearts. The clinical trial demonstrated the feasibility of using clinically safe hESC-derived progenitors, and the lack of any negative safety parameters during the trial enables the cells to be used for further clinical investigation.

In addition to these recent findings, we have previously described a stable and reproducible method for the differentiation of hESCs to cardiovascular progenitors (CVPs) at days 9 to 11 of our differentiation protocol: by culturing the stem cells on a humanized matrix surface comprising the heart-abundant laminin, LN221^13^. When transplanted into infarcted mice hearts, these CVPs were successfully engrafted into the infarcted area, where they led to graft vascularization and maturation, as well as heart function improvements over time. While promising, these results need to be validated in detail in terms of human cardiac muscle formation, heart rhythm, and functionality in a large animal model. Therefore, we have chosen the pig heart as a model for such studies because it is an animal model physiologically similar to the human heart^14^.

In the present study, we investigated approximately 200 million, day 11 committed cardiac progenitors (CCPs) for their potential to regenerate damaged heart tissue. Due to severe concerns that transplantation of beating CMs can cause lethal arrhythmia and poor functional improvements^2^, we chose to transplant non-contracting CCPs. The rationale for testing the later CCP timepoint comes from our previous study, where we observed a more organized human graft in day 11 cells as compared to the earlier day 9 cells^13^. Our present results suggest that CCPs have the potential to be developed into a safe cell therapy product for ischemic heart disease.

## Results

### Committed cardiac progenitors and their signature genes

**Fig. 1:**
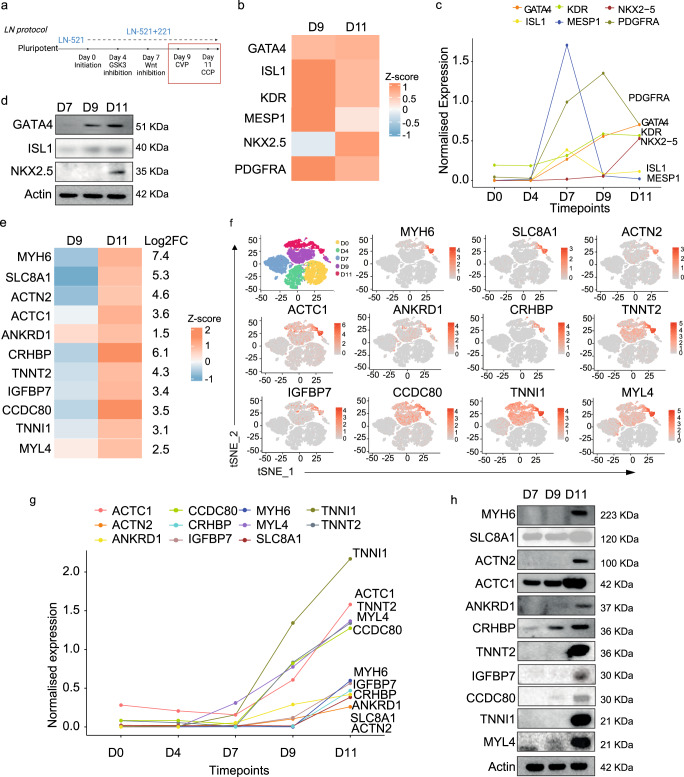
Committed Cardiac Progenitors and their Signature Genes. **a** Schematic showing the laminin (LN) protocol that uses LN-521 + 221 as a defined extracellular matrix together with small molecule inhibitors for the differentiation of pluripotent stem cells to day 11 committed cardiac progenitors (CCPs). **b** Heat map of three biological replicates from bulk RNA-seq data demonstrating the canonical progenitor gene expression on days 9 and 11. Supplementary Table 1 shows the actual transcripts per million (TPM) for each gene. **c** Time course analysis of scRNAseq data from days 0 to 11 for known canonical gene expression: *PDGFRA*, *GATA4*, *KDR*, *NKX2–5*, *ISL1*, and *MESP1*. **d** Immunoblotting of canonical progenitor genes from differentiation days 7, 9, and 11. Supplementary Fig. 1 shows the quantification of each blot from five biological replicates. **e** Heat map of the bulk RNA-seq data using CCP signature genes at differentiation days 9 and 11. Supplementary Table 1 shows the actual TPM for each gene. **f** Single-cell RNA-seq data (two biological replicates) of the CCP signature gene expression in tSNE plots from days 0, 4, 7, 9, and 11. The tSNE plot at the top left shows the overall distribution of each day using different colors. **g** Time course analysis of scRNAseq data from days 0 to 11 for day 11 CCP gene signature markers. **h** Immunoblotting of CCP signature genes from differentiation days 7, 9, and 11 (5 biological replicates). Supplementary Fig. 1 shows the quantification of each blot. Comparisons between groups were performed using two-way ANOVA with Tukey post hoc analysis.

Since cellular products are defined as drugs by the FDA and EMA^15^, we decided to explore if the cells generated in this study share homogeneous phenotypic characteristics and fulfill acceptable safety and efficacy requirements. We wished to ensure that the cells possess in vivo regeneration ability but lack tumorigenicity and that they exhibit similar transcriptomic biomarkers profiles. The differentiation protocol using an initial LN-521 + 221 matrix and the later addition of small molecule inhibitors that we employed to differentiate hESC into cardiac progenitors at days 7 to 11 has been reported previously^13^ (Fig. 1a).

There is a time-dependent expression of genes that are up-regulated over time as the hPSCs differentiate towards cardiac cells^16^. The earliest is the *ISL1*-expressing cardiovascular progenitors (CVPs), followed by the NKX2.5-expressing CCPs^17^. Using this knowledge, we have identified cells on day 7 as CVPs and cells on day 11 as CCPs. Therefore, we have chosen day 11 cells to investigate further in this study because we have observed previously that day 11 cells generated more mature human muscle fibers than day 9 cells^13^.

To define the CCP gene signature, we performed differential gene expression using bulk RNA sequencing (RNA-seq) between differentiation days 9 and 11 and obtained the set of genes typically expressed in day 11 CCPs. We observed that day 11 CCPs have lower expression of the previously reported canonical early progenitor marker genes (*ISL1*^18^, *KDR*^19^, *MESP1*^6^, and *PDGFRA*^20^) (Fig. 1b). The expression of genes *ISL1* and *MESP1* have a lower expression on day 11 than on day 9, but late progenitor genes such as *GATA4*^21^ *and NKX2.5*^22^, showed higher expression on day 11 as compared to day 9 which can be used to identify our cells as late progenitors.

To show the gene expression changes over time, we plotted the expression level of these reported canonical early progenitor markers and showed the changes as the cells differentiate (Fig. 1c). The *MESP1* and *ISL1* gene expression peaked on day 7 and rapidly decline on day 9, and *PDGFRA* expression peaked on day 9 and showed a downregulation on day 11. On one hand, *GATA4* and *KDR* did not show any significant increase in expression after day 9 when they appeared to have plateaued. On the other hand, we observed a rapid increase in *NKX2.5* expression from day 9. We are confident of our data because we have analyzed two biological replicates per day and observed a high degree of overlap between the spots in each replicate. This underpins the high reproducibility of our laminin-based protocol (Supplementary Fig. 1a).

Subsequently, we validated the RNA-seq results using quantitative PCR (qPCR) (Supplementary Fig. 1), and we also quantified the protein levels by immunoblotting (Fig. 1d and Supplementary Fig. 1c). Both approaches yielded similar results, i.e. the investigated genes *GATA4*, *ISL1*, *KDR*, *MESP1*, *and PDGFRA*, did not exhibit higher expression on day 11 when compared to day 9 (*p*-value > 0.05). An exception was *NKX2.5* (*p*-value < 0.05), which showed significantly higher expression on day 11. Therefore, we proceeded to search for additional biologically meaningful gene markers that would be more suitable for the characterization of the CCPs.

We used a time-course bulk RNA-seq dataset to search for genes that are highly expressed on day 11 and identified a gene signature (including *MYH6*, *SLC8A1*, *ACTN2*, *ACTC1*, *ANKRD1*, *CRHBP*, *TNNT2*, *IGFBP*, *CCDC80*, *TNNI1*, and *MYL4*) that could distinguish between day 9 and 11 CCPs (Fig. 1e). We observed an increase in log2 fold change of 1.5 to 7.4 fold on day 11 as compared to day 9, while most of the genes with an average log2 fold change of 3 folds, suggested the up-regulation of the genes on day 11. The genes were highly expressed on day 11 and maintained in their expression on day 20^23^ (Supplementary Fig. 1d, Supplementary Table 1).

To obtain a higher resolution of the expression data, we performed single-cell RNA-seq (scRNA-seq) on days 0, 4, 7, 9, and 11 (Fig. 1f). The t-distributed Stochastic Neighbor Embedding (tSNE) plots show a day-specific expression of each CCP marker gene. In tandem with our previous data, the plots showed higher expression of these genes on day 11 as compared to day 9. Therefore, to highlight our gene panel, we plotted a time-dynamic expression of these marker genes and observed their significant increase in the 11 CCP gene expression over time using our differentiation method (Fig. 1g).

Next, we validated the RNA-seq results by comparing the expression levels of the signature genes in different differentiation batches using qPCR (Supplementary Fig. 1e) and immunoblotting (Fig. 1h and S1f). As expected, most of the genes, except *ACTC1*, *CRHBP*, and *CCDC80*, showed a significant increase (*p*-value < 0.05) in the expression on day 11 compared to day 9. Therefore, this gene signature can be used as a quality control criterion for confirming the identity of day 11 CCPs, in each of the differentiation batches.

To further understand the cellular heterogeneity in the day 11 cells, we visualized the scRNAseq results from day 11 in a tSNE plot. A major population of CCP (33.2%) and mesenchymal and fibroblast (56.7%) and a smaller population of epithelial (8%) and endothelial (1.8%) cells were identified (Supplementary Fig. 1g). It is to be expected to have cellular heterogeneity in the cellular differentiation and we acknowledge the presence of a large proportion of mesenchymal and fibroblast clusters. It has been suggested that fibroblasts may play a role in the actual cardiac muscle regeneration and therefore, it could be beneficial to have them in the repair process^24^. However, further studies on the actual role of fibroblasts in muscle regeneration should be considered.

In addition to investigating the expression of the progenitor marker genes, we performed optical electrophysiological imaging to determine the electrophysiological status of these CCPs (Supplementary Fig. 2a, b). We were unable to detect membrane activity at day 11, but fluctuations in signals were observed between day 12 and 14, and the first detectable cardiac-like action potential waveforms were detected on day 15. Measurable membrane potentials were detected on day 17, with all three CM action potential traces (ventricular-like, atrial-like, and nodal-like CM).

Finally, to explore transplantation safety, we performed a teratoma formation assay in nude mice by injecting luciferase-labeled CCPs and pluripotent cells into the hindlimb (Supplementary Fig. 2c). These cells are stable after the lentivirus transduction as shown by karyotyping analysis made to ensure normal chromosomal numbers and no gross abnormality (Supplementary Fig. 2d). After eight weeks post-transplantation, we observed tumors in the mice injected with pluripotent cells but none in mice injected with differentiated CCPs. Since our CCPs are luciferase-labeled, we imaged the mice under the IVIS machine and identified their presence at the injection site. Altogether, these data show that the CCPs are viable and that they are unlikely to be tumorigenic.

### Vascularization of human muscle grafts

**Fig. 2:**
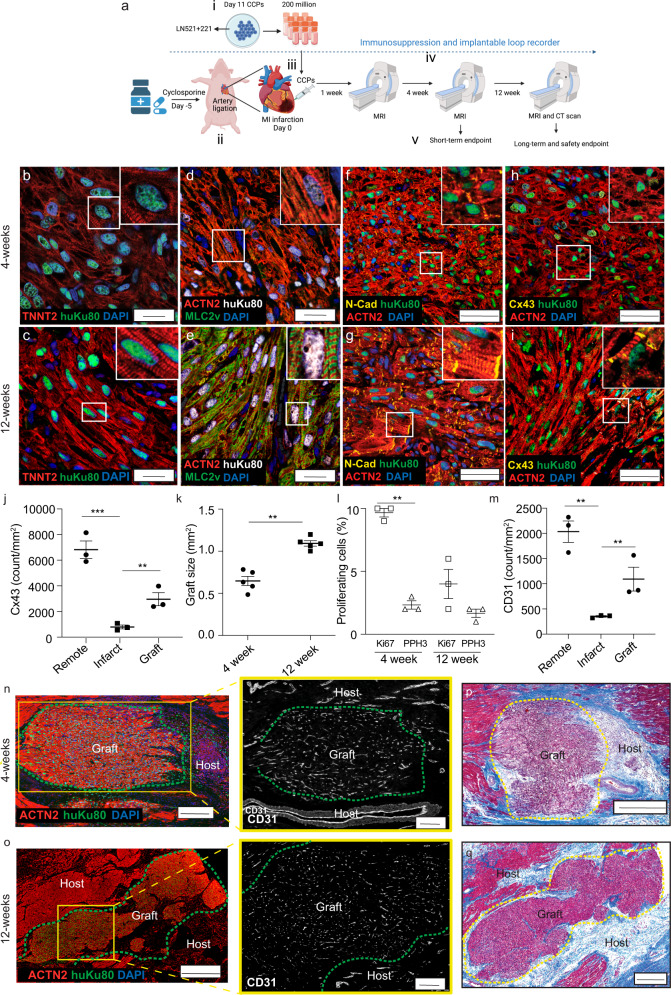
Vascularization of human muscle grafts. **a** Schematic illustration of: (i) CCP generation; (ii) permanent MI infarction; (iii) intramyocardial transplantation of CCP; (iv) post-transplantation MRI and CT scans, and (v) euthanization at 4 or 12 weeks post-transplantation. At 4 weeks, the human grafts showed disorganized staining of **b** TNNT2 (red) Scale bar = 20 μm, **d** ACTN2 (red) and MLC2v (green) Scale bar = 20 μm, **f** N-Cadherin (N-cad, yellow), and ACTN2 (red) scale bar = 50 μm and **h** connexin-43 (Cx43, yellow) and ACTN2 (red) Scale bar = 50 μm. At 12- weeks, human grafts show highly organized staining of **c** TNNT2 (red) scale bar = 20 μm, **e** ACTN2 (red) and MLC2v (green) scale bar = 20 μm, **g** N-Cad (yellow), and ACTN2 (red) Scale bar = 50 μm, and **i** Cx43 (yellow) and ACTN2 (red) Scale bar = 50 μm. **j** Quantifications of gap junction protein (Cx43) at the remote, infarct, and graft areas at 12 weeks. (***p-value < 0.0005, **p-value < 0.005). **k** Quantification of graft size by measuring the area of ACTN2 in the human graft in each 10x field of view. **p-value < 0.005, *n* = 5. **l** Quantification of images stained with proliferation markers Ki67 or PPH3. Refer to Supplementary Fig. 4f. **m** Quantification of blood vessels (CD31) at the remote, infarcted, and graft area at 12 weeks. (**p-value < 0.005, **p-value < 0.005) *n* = 3 and expressed as mean ± SEM. **n**, **o** Left: Confocal immunofluorescence images of 4- or 12- week post-transplantation with ACTN2 (red), huKu80 (green), and DAPI (blue). Scale bar = 100 μm. Right: The yellow box shows anti-CD31 (white) staining, Scale bar = 100 μm. **p**, **q** Masson trichrome staining of the transplanted region at 4- or 12 weeks (of **n** and **o** respectively) shows a low amount of collagen deposition (blue) around the human graft (purple) and host tissue at the edges (dark red). Scale bar = 500 μm and 1 mm Scale bar = 1 mm. Refer to Supplementary Fig. 4.

All the pigs received immunosuppression five days prior to the transplantation of the CCP cells. The treatment was maintained throughout the four- or eight-week experiments (Fig. 2a); Supplementary Fig. 3 depicts a summary flow chart of the experiment. Immunohistology staining in the human muscle grafts using immune cell-specific markers CD45, CD20, and CD3 showed the absence of pan-leukocytes, B-lymphocytes, and T-lymphocytes, respectively (Supplementary Fig. 4a). However, three pigs from the medium-control group and two pigs from the CCP transplanted group developed complications from immunosuppression, such as anemia or lung infection (refer to Supplementary Fig. 3 and Supplementary Table 3 for details).

At the end of 12 weeks, the pigs were euthanized, and their hearts were excised. The ligation sutures were visible and intact on the day of euthanization (Supplementary Fig. 4b). The viability of transplanted cells at the infarction site could be monitored using live in vivo IVIS optical imaging following the administration of luciferin into the coronary artery at the time of euthanization (Supplementary Fig. 4c). The regions of heart muscle fibers with positive luciferase signals were identified and processed for histology staining. Hematoxylin and Eosin (H&E) staining showed large human grafts at the peri-infarct area close to the pig’s normal muscle tissue (Supplementary Fig. 4d).

The identity of the grafts was confirmed by immunohistology staining with a human nucleus-specific antibody, cardiac troponin T (TNNT2), alpha-actinin-2 (ACTN2), and myosin light chain 2 V (MLC2v) at 4- and 12 weeks (Fig. 2b–e and Supplementary Fig. 4e). We observed Ku80-positive (Ku80 +) human cardiac muscle grafts with highly organized sarcomere striations as the graft matured. Alternate ACTN2 and MLC2v striations were initially absent at 4 weeks (Fig. 2d), with the proper alignment becoming apparent at 12 weeks (Fig. 2e), which suggested the maturation of the human graft. It is noteworthy that almost all the human cells expressed ventricular-like MLC2v instead of atrial-like MLC2a (Supplementary Fig. 4e), indicating that most of the cells are ventricular and not atrial CMs.

N-Cadherin (N-Cad) expression was examined to determine the presence of cell-to-cell connections in the human grafts (Fig. 2f–g). We observed only weak expression and spotty distribution of N-Cad staining at 4 weeks, but the protein became better organized and highly expressed at 12 weeks. Thereafter, we determined the expression of connexin-43 (Cx43), a major electrical determinant of the electrical properties of the gap junctions in human CMs^25^. We observed similar but weaker Cx43 expression at 4 weeks, following which the protein exhibited stronger expression and highly organized distribution by 12 weeks (Fig. 2h, i). Cx43 quantification in 3 different areas (remote, infarct, and graft) at 12 weeks showed significant expression of the protein in the graft as compared to the infarction site (*p*-value < 0.05) (Fig. 2j). As Cx43 regulates intercellular coupling and conduction velocity, the results suggest that the human grafts continue to mature and become electrically coupled and physiologically functional over time^25^.

We then determined the graft size by quantifying human ACTN2 at 4- (0.6 ± 0.1 mm^2^) and 12 (1.1 ± 0.1 mm^2^) weeks (Fig. 2k). The results showed a significant increase in human graft size (*p*-value < 0.05) across time. The proliferation capacity of the cells was also determined using the proliferation markers, Ki67, and phospho-histone H3 (PPH3) to quantify the proportion of proliferating cells at 4 and 12 weeks post-transplantation (Fig. 2l and Supplementary Fig. 4f). At 4 weeks, 9.7 ± 0.3% of the cells were positive for Ki67 and about 2.3 ± 0.3% were positive for PPH3 (*p*-value < 0.05). In contrast, there was a significant reduction in the proliferation rate at 12 weeks, with only 4 ± 1.2% and 1.7 ± 0.3% cells being Ki67-positive and PPH3-positive, respectively. The results suggest that the differentiated CMs derived from the CCPs are proliferative at four weeks and gradually become less proliferative cells in the regenerated heart muscle.

Rapid engraftment, with new heart tissue growth and functional development, was observed as early as at 4 weeks. Since this regeneration process cannot occur without a sufficient blood supply, we analyzed the vascularization of the human grafts by staining the tissue for the endothelial marker CD31 at 12 weeks (Figs. 2m, o). CD31 quantification in the remote infarcted and grafted areas showed that blood supply in the remote area was impaired after MI (*p*-value < 0.05), but that an extensive amount of blood vessels was regenerated in the graft area as compared to the infarcted area. We measured a significant 125% increase in blood vessel generation in graft as compared to the infarcted area (*p*-value < 0.05, Fig. 2m).

Histology sections stained with Ku80, ACTN2, and CD31 at 4- and 12 weeks revealed the size of the graft and the underlying CD31-positive blood vessels (Fig. 2n, o). The yellow box (bottom) shows a magnified view of the human graft. Since these blood vessels were not positive for human-Ku80, we can speculate that they could be derived from the collateral blood vessel network of the host myocardium surrounding the scar.

Masson trichrome staining was used to visualize collagen fibers in the scar tissue. We showed that the area surrounding the scar has less collagen deposition (*p*-value < 0.05) (Fig. 2p, q) compared with medium-control hearts (Supplementary Fig. 4g, h). The presence of micro-vessels, associated with a reduction in fibrosis, suggests that the CCPs can have chemoattractive properties that stimulate the growth of new blood vessels and reduce scarring.

Other cell types in the graft were also examined. We stained the infarcted region for the presence of endothelial CD31 or vimentin (VIM) for fibroblasts together with the human-specific Ku80 antibody (Supplementary Fig. 4i). We observed the presence of human CD31+ blood vessels and VIM+ fibroblast-like cells in the human graft. This shows that the transplanted CCPs are associated with other cell types.

### Improvement in heart function

**Fig. 3:**
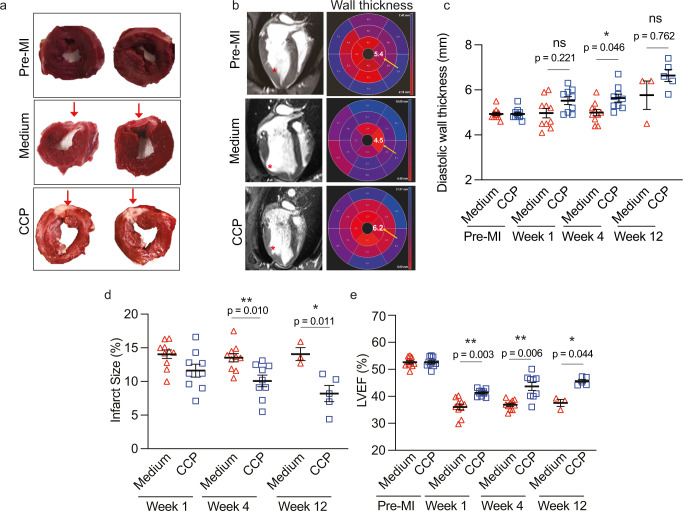
Improvement in heart function. **a** Representative transverse section of two independent pre-MI, medium, and CCP-transplanted left ventricles at 12 weeks post-transplantation. The transmural infarct region is shown in pinkish white (red arrows) with obvious ventricle remodeling in medium-control as compared to CCP-transplanted hearts. **b** Left: 4-chambers long axis MRI slices from pre-MI, medium, and CCP-transplanted hearts at 12 weeks post-transplantation. Significant adverse ventricular remodeling in medium-control hearts as compared to pre-MI and CCP-transplanted hearts (red asterisks). Right: Representative polar plots (in AHA scale) of left ventricle wall thickness segmentations in pre-MI, medium, and CCP-transplanted hearts. Functional values in the apical lateral segment are highlighted with a yellow arrow. **c** Quantification of diastolic wall thickness in AHA scale at 1, 4, and 12 weeks post-transplantation. Wall thickness in CCP-transplanted hearts was significantly greater than in medium-control hearts at 4 weeks (*p-value = 0.0046). Refer to (**a** and **b**) for representative images. **d** Infarct size was measured at 1, 4, and 12 weeks post-transplantation. Infarct size was significantly reduced in CCP-transplanted hearts as compared to medium-control hearts at 4 and 12 weeks. (**p-value = 0.010, *p-value = 0.011). **e** Left ventricle ejection fraction (LVEF) was determined and measured across 1, 4, and 12 weeks post-transplantation. Significantly higher LVEF was observed in CCP-transplanted hearts as compared to medium-control hearts at 1 (**p-value = 0.003), 4 (**p-value = 0.006) and 12 weeks (*p-value = 0.044).

At the end of 12 weeks, the pigs were subjected to magnetic resonance imaging (MRI) and computerized tomography (CT) scans to determine heart function and long-term safety profile (*n* = 9–10 at 1 and 4 weeks, *n* = 3–5 at 12 weeks) (Supplementary Table 3). The left ventricles (LV) were cross-sectioned into five rings from the apex to the atrium. We observed gross morphology differences in the pre-MI, medium, and CCP-transplanted hearts (Fig. 3a). While pre-MI was an uninjured LV with healthy ventricular walls, medium-control pigs exhibited LV transmural scarring and adverse ventricular remodeling (red arrows), and CCP-transplanted pigs revealed lateral LV wall scarring (red arrows) without obvious ventricular wall thinning. These observations were independently confirmed by MRI scans (Fig. 3b, left panel). By using the 4-chamber long-axis view in the MRI scans, we could display the lateral wall thickness (red asterisks) in polar maps according to American Heart Association (AHA) segmentation guidelines^26^. The medium-control hearts showed reduced wall thickness in apical lateral (middle panel, yellow arrow) or mid-anterolateral segments as compared to CCP-transplanted hearts (Fig. 3b, right panel). The maps also showed that the diastolic wall thickness was similar at 1 week (*p*-value = 0.221), followed by a significant improvement at 4 weeks (*p*-value = 0.046) although the improvements were not sustained at 12 weeks (*p*-value = 0.762) (Fig. 3c and Supplementary Table 3). The loss of diastolic wall thickness at 12 weeks could possibly be due to the low number of replicates (*n* = 3 for medium control) at 12 weeks as compared to the other weeks (*n* = 10 for medium control).

We determined LV infarction size by MRI measurements (Fig. 3d, Supplementary Table 3). Initially, at 1-week, the infarction size of 12–15% was similar in both medium-control and CCP-transplanted hearts. As the scar developed further between 4- and 12 weeks, there was a significant reduction in infarction scar size from 13 ± 1% in medium-control to 10 ± 0.8% at 4 weeks (*p*-value = 0.01) and to 8 ± 1.2% at 12 weeks (*p*-value = 0.011). This suggests that progressive regeneration of the infarcted muscle tissue occurs by the transplanted human CCPs.

As expected, both medium-treated and CCP-transplanted hearts had reduced Left Ventricle Ejection Fraction (LVEF) (medium: 36 ± 1.1%, CCP: 41 ± 0.4%) as compared to pre-MI hearts (pre-MI: 53 ± 0.6%), at 1-week post-transplantation (Fig. 3e). Surprisingly, there was significant improvement (*p*-value = 0.003) in medium- (36 ± 1.1%) and CCP-treated (41 ± 0.4%) hearts as early as at 1-week. A similar significant improvement was observed at 4 weeks (medium: 37 ± 0.6%, CCP: 44 ± 1.5%, *p*-value = 0.006) and 12 weeks (medium: 38 ± 1.3%, CCP: 46 ± 0.7%, *p*-value = 0.044) suggesting that the transplanted CCPs contribute to the improvement in LVEF.

Collectively, these results suggest that the lateral ventricular wall was adversely affected by MI, with obvious wall thinning, and a reduction in ejection fraction. However, these changes were in part restored in hearts transplanted with CCPs at 12 weeks post-transplantation.

A significant concern about using pluripotent stem cells in regenerative medicine is the potential development of tumors^27^. We investigated the in vivo safety of transplanted cells in the pigs by performing computerized tomography (CT) scans at 12 weeks post-transplantation. The results revealed no signs of visible tumors growth in any of the animals after cell transplantation (Table 1).

**Table 1:** Pigs characteristics and summary data.

ID	Weight (kg)	Sex	Treatment	End-point	Electrophysiology analysis	Presence of VT?	CT findings
**2515**	13	M	Media	4-weeks	4 weeks	No	NA
**2545**	12.6	F	Media	4-weeks	NA	No	NA
**2548**	12	M	Media	4-weeks	NA	No	NA
**2666**	10.6	M	Media	4-weeks	NA	No	NA
**2668**	11.8	F	Media	7-weeks	NA	No	NA
**2665**	10	M	Media	8-weeks	NA	No	NA
**2585**	11.8	F	Media	9-weeks	2 weeks	No	NA
**2517**	15	M	Media	12-weeks	NA	No	NA
**2572**	11.6	M	Media	12-weeks	NA	No	6 mm nodule in the right upper pulmonary lobe, no calcification, indetermined;
**2583**	12.8	M	Media	12-weeks	2 weeks	No	No tumors
**2469**	10.82	F	D11	4-weeks	NA	No	No tumors
**2520**	13	M	D11	4-weeks	NA	No	No tumors
**2584**	13.1	F	D11	4-weeks	2 weeks	No	No tumors
**2578**	11.86	M	D11	10-weeks	NA	Yes	No tumors
**2576**	12	M	D11	10-weeks	NA	Yes	No tumors
**2498**	13.4	F	D11	12-weeks	NA	Yes	No tumors
**2519**	13.6	M	D11	12-weeks	4 weeks	No	No tumors
**2577**	12.8	M	D11	12-weeks	2 weeks	Yes	No tumors
**2573**	12	F	D11	12-weeks	2 weeks	Yes	No tumors
**2673**	12.6	M	D11	12-weeks	NA	No	No tumors

### Temporary ventricular arrhythmia in 50% of CCP-transplanted pigs

Accurate discrimination between healthy and infarcted myocardia is crucial in assessing the efficacy of CCP transplantation. Electromechanical (EP) mapping with the 3D-NOGA system can simultaneously register the electrical and mechanical activities of the left ventricle, enabling in vivo assessment of myocardial viability. Here, we used the NOGA system to identify and localize potential arrhythmogenic foci and direct therapeutic procedures.

**Fig. 4:**
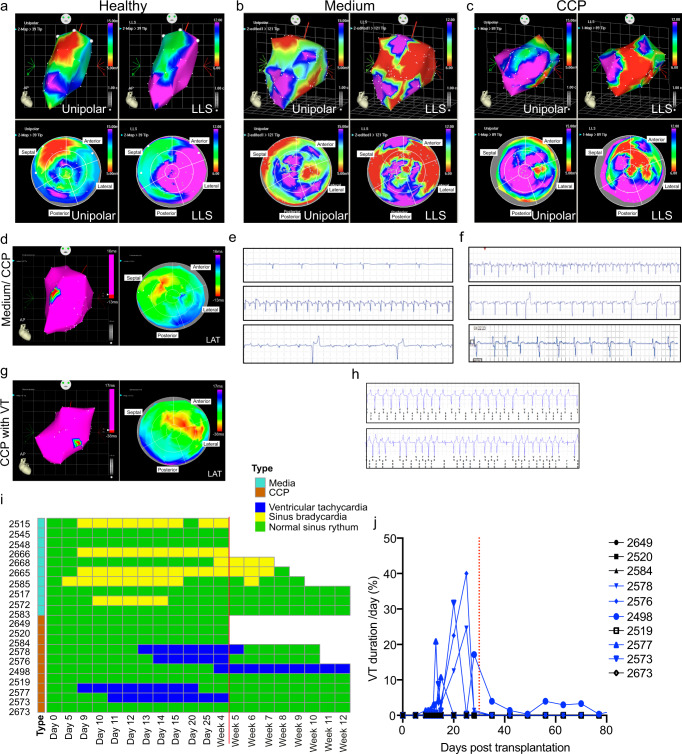
Temporary ventricular arrhythmia in 50% of CCP-transplanted pigs. Lateral left ventricle endocardial voltage map. **a** Healthy uninjured ventricle, **b** MI with medium injection, and **c** MI with CCP-transplantation. Unipolar voltages lower than 5 mV are considered scar (red color), and healthy and viable myocardium are indicated as purple. A local linear shortening (LLS) map provides information about wall movement (red = low movement, purple = healthy movement). Overlapping regions with scars (unipolar map) and non-contracting areas (LLS map) are seen. See Table 1 and Supplementary Table 4. **d** (Left) Electro-anatomical maps in medium-control or CCP-transplanted pigs at 2 weeks post-transplantation. The map of a healthy LV without ventricular tachycardia is shown. (right) The local activation time (LAT) map shows LV contraction of a normal electrical activation wave-front. **e** Ventricular tachycardia (VT) was observed in two of the CCP-transplanted pigs at 2 weeks post-transplantation. (left) Electro-anatomical maps of VT occurring in CCP-transplanted pigs at 2 weeks post-transplantation. The map of an injured LV with VT is shown (right). The local activation time (LAT) map shows LV contraction of an abnormal electrical activation wave-front during VT. **f** Representative ECG traces from the implantable cardiac monitor of medium-control hearts showed (top) sinus bradycardia, (middle) sinus tachycardia with ST elevation, and (bottom) premature ventricular contraction. **g** Representative ECG traces from the implantable cardiac monitor of CCP-transplanted hearts (without VT) showing (top) sinus tachycardia, (middle) premature ventricular contraction, and (bottom) atrial fibrillation. **h** Representative ECG traces from the implantable cardiac monitor of CCP-transplanted hearts (with VT). **i** Heat map showing the overall time course analysis from the implantable cardiac monitor. **j** Graph showing the quantification of VT duration per day in CCP-transplanted pigs. Pigs with normal sinus heart rhythm are displayed in black and pigs with VT are shown in blue. The red dotted line shows the 4-week timeline when VT in 4 of the pigs resolved compared to one pig (#2498) where it did not.

Several pigs underwent the EP mapping procedure at 2 and 4 weeks (Supplementary Table 4). Unipolar values (mV) provide information on the viability of the tissue by detecting the voltages across the myocardium, while local linear shortening (LLS) values provide information on wall movement. The anterior and lateral walls will be affected by the ligation of one of the blood vessel branches of the LAD and LCX arteries. In our experiments, the healthy pigs exhibited a healthy voltage map as shown using unipolar and LLS values in the anterior and lateral walls (Fig. 4a). The results were displayed using LV voltage (top panels) and bull’s eye polar (bottom panels) maps. The absence of low voltage segments indicating healthy myocardium (pink areas) is apparent. In contrast, the media control hearts exhibited segments with low voltages and low movement (red area) in the unipolar and LLS map (Fig. 4b, top panels). As expected, the bull’s eye polar map demonstrates that the low voltage segments (red areas) and low wall movement (red areas) are at the anterior and lateral walls (Fig. 4b, bottom panels). It is noteworthy that overlapping of the low voltage segments and reduced wall movement at the lateral wall are reliable indicators of a scar. The electrophysiological (EP) property of the LV in CCP-transplanted pigs was subsequently determined (Fig. 4c). At 2 weeks, we observed similar low unipolar voltages (red areas) and low wall movement (red areas) in the anterior and lateral walls, although to a lesser extent. There was also an overlap of unipolar and LLS segments, indicating the presence of a scar. In parallel, the results from the infarct size measurements using MRI measurements from all the pigs (*n* = 9–10 at 1 and 4 weeks, *n* = 3–5 at 12 weeks) showed a significant reduction in infarct size at 4 and 12 weeks (Fig. 3e).

To understand the relation between electrical connectivity and heart rhythm, electroanatomic mapping (EAM) was carried out on the pig heart. The medium-control pigs showed normal electrical activation (Fig. 4d, left panel). The local activation map (Fig. 4d, right panel) and propagation map (Supplementary Video 1) showed the earliest electrical activation (red areas) starting from the septum consistent with descending activation from the left bundle branch with the latest activation (blue areas) occurring at the lateral segment as expected. The medium-control pigs exhibited normal electrical conductance without ventricular tachyarrhythmia (VT) as confirmed with implantable telemetric ECG recorders (Fig. 4e). These traces were blindly analyzed by a clinical electrophysiologist who reported the presence of numerous episodes of sinus bradycardia (top), sinus tachycardia with ST elevation (middle), and premature ventricular contraction (bottom) but no VT.

Analyses of CCP-transplanted hearts at 2 weeks showed that the pigs had similar electrical activation as medium-control pigs. Initial activation started at the septum and ended at the lateral wall. We also observed conduction into the scar containing human grafts, which suggested electrical conductivity between the host and engrafted cells (Fig. 4d, Supplementary Video 2). Typical ECG traces from the cardiac monitor in CCP-transplanted pigs that did not develop VT showed sinus tachycardia (top), sinus rhythm with premature ventricular contractions (middle), and atrial fibrillation (bottom) (Fig. 4f). However, we observed two CCP-transplanted pigs that developed focal VT, confirmed by both the NOGA machine and implantable cardiac monitor. The earliest activation (red areas) started at the lateral wall and the latest activation ended at the posterior segments (blue areas) of the LAT polar map (Fig. 4g, right panel, and Supplementary Video 3). Typical VT traces from the cardiac monitor in CCP-transplanted pigs, that developed VT, are shown in Fig. 4h.

Even though EP mapping was not performed on all the pigs, all 10 pigs were implanted with telemetric cardiac monitors (implantable loop recorders, ILR) enabling the detection of VT (Table 1). These ILRs trigger data collection when pre-set criteria are met, so episodes of possible VT are recorded. Despite known limitations of this monitoring system (discussed in Methods), we observed that five out of ten CCP-transplanted pigs developed VT. However, the VT was temporary, developing at around 10 days post-transplantation and lasting for ~30 days, after which the VT resolved by itself, and the heart returned to normal sinus rhythm (Fig. 4i). Quantification of the duration of VT per day to show VT burden was plotted as shown in Fig. 4j. The results show that in the first 30 days, the four pigs (# 2578, #2576, #2577, and #2673) underwent VT episodes from 1 to 40% (14 min to 9.6 h) of the time in a day. Subsequently, the duration of VT diminished rapidly and remained absent throughout the experiment. The absence of VT in the remaining five pigs (#2649, #2520, #2584, #2519 and #2673) represents a significant improvement from previous studies in large animals treated with contracting human CMs^5,28,29^, which all exhibited extensive arrhythmia and VT-related death. Importantly, we did not observe persistent VT in but one (#2498) out of the ten pigs tested. In this animal, VT episodes started at approximately 4 weeks post-transplantation at 17% (4 h) of VT burden per day and continued until the end of the experiment without any resolution at about 1–4% (14.4 mins to 1 h) duration per day (Fig. 4j). Nevertheless, despite the persistence of long-lasting episode of VT, this pig did not die of fatal VT at the end of 12 weeks when the experiment ended. Conclusively, these results suggest that non-contracting CCPs have the potential to be developed into safe means for cell therapy.

## Discussion

The results of this study suggest that non-contracting hESC-derived CCPs can possibly be developed into a safe method for stem cell based cellular therapy of MI. Importantly, our study suggests that the stable and reproducible laminin-based method for differentiation of CCPs from hESCs can be applied to regenerative cardiology. In addition to using LN521 + 221 to generate CVPs and CCPs^13^, we have previously shown that differentiation of hPSCs on human biologically relevant recombinant laminin matrices is highly reproducible, e.g. for making endothelial cells (LN521)^30^, photoreceptor progenitors (LN523)^31^, and human keratinocyte stem cells (LN211)^32^. In addition, Parmar’s group has shown that hPSCs differentiated on LN-111, that is an abundant ECM protein in the midbrain, differentiate to dopamine neurons^33^. The differentiation consistency and reproducibility obtained on laminin matrices is usually not achieved in protocols using variable and undefined cellular substrates, such as feeder cells or the mouse tumor extract Matrigel®^34,35^. We and others advocate the use of stable and reproducible differentiation methods for the development of successful hPSC-based cell therapy methods^2^.

Following transplantation into the infarcted heart region, the CCPs rapidly organize themselves in the scar, generate a human heart muscle graft in the host heart, and continue to mature to form critical Cx43 connections (Fig. 2) necessary for the development of the heart muscle. Although we report findings similar to those described previously^5,11,36–39^, our method differs in its use of approximately 200 million CCPs to improve heart function after MI, which we consider an improvement when compared to previous methods, which required approximately 750 million to one billion CMs in pigs and macaques^5,37^. The mechanistic basis of the proliferation and efficacy of CCPs remains unclear, but it could be attributed to differences in cell potency between CMs and CCPs, as the former are terminally differentiated while the latter are not^40–42^. It is possible that these transplanted CCPs offer beneficial effects through indirect mechanisms involving the exosomes and micro RNAs^43^. Further studies will be performed to confirm our hypothesis and elucidate the possible cardiac regeneration mechanisms. Interestingly, a recent study by Poch et al. reported that human cardiac progenitors facilitate CXCL12/CXCR4-dependent homing to injury sites, and SLIT2/ROBO1 signaling targeted fibrosis which leads to functional restoration of damaged heart muscle^44^. We explored the expression of *CXCL12* and *CXCR4* in our 1-week transcriptomic data from the human graft (unpublished data). However, we were unable to detect any gene expression in the graft. We reason that since the CCPs are directly injected into the infarcted region, our cells will not require CXCL12/CXCR4 signaling for migration. We also looked for *SLIT2* and *ROBO1* gene expression in our graft and similarly, we did not observe any expression. It is possible that the repulsion of fibroblasts by the progenitors has occurred before week one and thus we are unable to measure its expression or there could be other repair mechanisms that need to be uncovered.

While we acknowledge that similar studies^5,7,8,37,39^ have been reported before, our study differs from others in terms of the MI model used (permanent ligation vs ischemia-reperfusion), the timing of cell administration (immediately post-MI vs weeks later), cell dosage (200 million vs 1 billion), and frequency of ECG monitoring (daily vs time-points). In the present study, we compared to pre-MI hearts and observed a significant improvement of LVEF in CCP-transplanted hearts at 4- and 12 weeks post-transplantation. In contrast, Laflamme’s group observed no significant LVEF improvements in CM-transplanted pig hearts compared to the medium controls^5,37^.

A critical problem that has surfaced in regenerative cardiology studies is the development of VT in pigs and NHPs that have been transplanted with immature CMs into the infarcted region^5,37^. We compared the present study against another one by Romagnuolo et al.^5^, that involved the transplantation of 1 billion contracting CMs into pigs 3 weeks after balloon occlusion of mid-LAD. While the two studies use different MI models (acute versus chronic), cell dosage, and timing of cell administration, both approaches use the same animal model and similar endocardial transplantation of cells. Here, we generated MI and transplanted 200 million CCPs into 10 pigs: about 50% of the pigs developed temporary VT at 0 to 4 weeks, and the frequency of VT fell sharply to 10% at 5 to 12 weeks. Importantly, no lethality occurred in our pigs due to VT. In contrast, Romagnuolo et al. generated CM transplantation in 6 MI-induced pigs and 100% of the pigs developed VT at 0 to 4 weeks; two pigs died of VT (33.3% VT-fatality). In an effort to prevent VT post-transplantation, Nakamura et al. recently reported the use of pharmacological therapy to effectively suppress arrhythmia, which could be a viable strategy to improve cellular therapy for heart regeneration^7^. The use of improved immunosuppressive drug regimens and other supportive medications will be considered in our future work.

Furthermore, we are uncertain why only five out of ten CCP-transplanted animals developed VT since all our transplanted animals had successfully engrafted human cells. We speculate that the occurrence of transient VT could be due to the animal model or surgery technique employed, or the presence of certain cells in the CCP population showing spontaneous depolarization or micro re-entry which is common in post-MI scarred heart. Further studies will be needed to unravel the mechanisms of VT following CCP transplantation.

To conclude, we place this study into the context of ongoing stem cell-based clinical trials for heart diseases. All the clinical trials using adult stem cell sources such as: skeletal myoblasts (MAGIC), bone marrow-derived mononuclear cells (BAMI, REGENERATE-AMI), mesenchymal stem cells (PROCHYMAL, RELIEF), hematopoietic stem cells (REGENT, COMPARE_AMI) and cardiac stem cells (SCIPIO, CADUCEUS), have demonstrated modest heart function improvements, the need for autologous transplantation and a lack of standardized study methodologies, thus limiting their clinical application^45^. On the other hand, clinical trials employing pluripotent stem cell sources such as induced pluripotent stem cells (iPSC)-derived cells have not been approved yet, while the ESCORT trial employing human embryonic stem cell (hESC)-derived progenitors reported 10% heart function improvements in severe heart failure patients.

Overall, we propose a laminin-based differentiation protocol as a mean to develop stem cell-based therapeutic strategies that could reproducibly generate sufficient and well-defined CCPs from allogenic sources. These cells have the potential to improve heart function and reduce VT episodes in infarcted hearts by 50%. However, future research will need to address additional challenges, including overcoming ethical and regulatory dilemmas to employ ESCs in humans.

There are limitations to the study. Firstly, the low number of replicates (*n* = 3, medium and *n* = 5, CCP) at 12 weeks needs to be increased to enhance the robustness of the results. Secondly, there is a lack of cardiac functional data at earlier time points (2–3 days) post-MI to ensure a similar reduction in LVEF to evaluate the impact of CCPs on regeneration as opposed to smaller infarct size. Thirdly, there is a limitation in the acute permanently ligated MI model. As permanent ligation may not reflect the majority of the potential patients that undergo reperfusion injury. Rather strategies in selecting chronic MI patients with large infarcts and LV dysfunction post-reperfusion will be more suitable. Therefore, we plan to explore the chronic MI model by reperfusion injury where we will measure the LVEF to ensure a similar reduction in LVEF before cell transplantation of the cells in the future.

## Methods

### Ethical statement

All human pluripotent stem cell studies were carried out with approval from the National University of Singapore’s Institutional Review Board (IRB 12–451). All the procedures involving animal experiments were performed with prior approval and following the protocols and guidelines of SingHealth’s Institutional Animal Care and Use Committee (IACUC) (2018/SHS/1426).

### Maintenance of human embryonic stem cells (hESCs)

All human pluripotent stem cell studies were carried out in accordance with approval from the National University of Singapore’s Institutional Review Board (IRB 12–451). Pluripotent hESCs H1 (karyotype: 46, XY; WiCell Research Institute, WA01, RRID:CVCL_9771) were maintained on 10 μg/ml LN521 (Biolamina AB, LN521)-coated culture plates with daily change of Nutristem® (Biological Industries, 05-100-1 A) medium. Routine monitoring of pluripotent markers POU5F1 (Santa Cruz, sc-5279, RRID:AB_628051) and Tra1–60 (Millipore, MAB4360, RRID:AB_2119183) by flow cytometry and genomic stability by karyotyping were performed at the Singapore General Hospital cytogenetics laboratory. Cells were split at 200,000 cells per well and were passaged at 80% confluence by gentle dissociation with TrypLE (ThermoFisher, 12563011) at 37 °C for 8 mins to dissociate the single cells. The cell suspension was then collected and centrifuged at 800 rpm for 4 mins. Supernatants were discarded and the cell pellets were resuspended in 1 mL of pre-warm Nutristem® medium. Bright-field images were taken with a Leica microscope.

### Committed cardiac progenitor differentiation protocol

Pluripotent cells were seeded at 6 million cells into 10-cm^2^ dishes (ThermoFisher, 150464) coated with combination matrices of (21.75 μg) LN-521 and (65.25 μg) LN-221 on day 0 in 5 ml of PBS. This protocol was based on our previous study^1^. At confluence (day 4), the cells were exposed to differentiation medium (RPMI 1640 (ThermoFisher, 11875-093) with B27 supplement without insulin (ThermoFisher, A1895601) and 10 μM of CHIR99021 (Tocris, 4423) for 24 h. The next day (day 5), the medium was removed and replaced with a differentiation medium. On day 7, the medium was changed to a differentiation medium with the addition of 5 μM of IWP2 (Tocris, 3533) for 2 days. Wells was replaced with a differentiation medium on day 9 and day 11. A 10-cm^2^ dish can generate ~20 million cells. Therefore, in order to achieve 200 million cells for 1 pig, we performed differentiation in ten 10-cm^2^ dishes. A schematic presentation of the differentiation protocol is shown in Fig. 1a.

### Generation of bulk RNAseq library

Bulk RNA sequencing data were generated in our previous publication^1^. Three bulk RNA-seq experiments were performed to assess the effect of LN-221. In this experiment, we sequenced pluripotent H1 cells growing on the combination of laminin matrices LN-521 and LN-521 + LN-221. The cells were harvested on days 0, 4, 7, 11, and 20. RNA sequencing libraries were constructed from total RNA using the Illumina Truseq Stranded Total RNA library preparation kit (with Ribozero Gold) and sequenced on Illumina HiSeq 3000 for three biological replicates at each time point. Reads are pair-ended with 150 bp length. RNA-seq reads were assessed for quality, aligned to GRCh38.79 using STAR 2.5.2b, and quantified with featureCounts. Ribosomal genes (Ensembl gene biotype “rRNA”) and mitochondrial genes were removed. The gene-level transcripts per million reads were computed from counts data. The selected CCP markers expression heatmaps were generated using ComplexHeatmap R Bioconductor package (v2.6.2).

### Time-series single-cell RNA profiling of cardiomyocyte differentiation

We generated a time-series single-cell RNA-sequencing dataset for cells derived from H1 hESCs using the laminin protocol (LN521 + LN221; Fig. 1) on days 0, 4, 7 9, and 11. We generated data from 2 biological replicates each day. A chromium instrument (10x Genomics) was used to partition viable cell suspensions into single-cell droplets using Single Cell 3’ library and gel bead kit version 2 (10x Genomics, Cat.#120237) as per manufacturer’s protocol. The samples were loaded into a single cell chip (10x Genomics, Cat. No.120236) to capture 3000 cells in each of the four sets of cells and sequenced using the Illumina Hi-Seq3000 sequencing platform in a single sequencing lane (Eurofins). The reads were aligned using CellRanger. The output matrices (i.e. matrix.mtx, genes.tsv and barcodes.tsv) generated from the alignment step were subjected to quality control filtering. Cells with very low and very high library sizes (i.e., cells below and above the 5^th^ and 99^th^ percentiles of the total cell library size respectively), cells with a low number of detected genes, and cells with more than 10% of their total gene count coming from mitochondrial genes were removed. Only the cell and genes that passed the quality control steps were considered for further analyses using the Seurat V3 pipeline. UMI counts were normalized by regularized negative binomial regression by using the *ScaleData* function with a default number of variable genes and by regressing the cell cycle genes (G2M and G2 phases). The RunPCA computed 50 principal components by considering just the variable genes. Next, *findElbow* function from the *ChemoSpecMarkeR* R package was run to estimate the number of principal components that could be used for t-distributed Stochastic Neighbour Embedding (tSNE) by using the elbow method. tSNE was run using the *RunTSNE* function from Seurat package. *FeaturePlot* was used to color the expression level of marker genes.

### Quantitative real-time polymerase chain reaction (qPCR)

Total RNA from Day 7, Day 9, and Day 11 was isolated using the Total RNA Purification Kit (Norgen Biotek, 17200) and quantified on the NanoDrop 1000 Spectrophotometer (Thermo Fisher Scientific). Complementary DNA (cDNA) synthesis from 5 μg of total RNA was performed using iSCRIPT^TM^ Reverse Transcription Supermix (BIO-RAD, 1708841) and T100 Thermal Cycler (Bio-Rad). Quantitative Real-Time PCRs (qPCR) were performed with the CFX384 Real-Time PCR system (BIO-RAD), using a total volume per reaction of 10 μl containing 1X SYBR^TM^ Select Master Mix for CFX (Applied Biosystems^TM^, 4472942), 2.5 μl of 1 μg cDNA template, 1 μl of 10 μM forward and reverse primers mix and 1.5 μl sterile RNase-free water. The following thermal profile was applied: 1 cycle at 95 ^o^C for 3 min, 40 cycles at 95 ^o^C for 10 sec and 60 ^o^C for 1 min, melt curve cycle from 65 ^o^C to 95 ^o^C for 2.5 min. Direct detection of PCR products was monitored by measuring the fluorescence produced due to SYBR Green dye binding to dsDNA after every cycle. The nucleotide sequences of forward and reverse primers for *MECR, GATA4, ISL1, KDR, KIT, MESP1, NKX2.5, PDGFRA, MYH6, SLC8A1, ACTN2, ACTC1, ANKRD1, CRHBP, TNNT2, IGFBP7, CCDC80, TNNI1, MYL4*, were tabulated in Supplementary Table 2. The Cq values were normalized to MECR as a housekeeping gene and results were plotted as relative expression units.

### Western blot

Cells were dissociated with TrypLE^TM^ Select Enzyme (Gibco, 12563011), centrifuged into a pellet in a microcentrifuge tube, and kept at −80 °C until all time points (days 7, 9, and 11) were collected. Cell pellets were washed once in ice-cold PBS and total protein was harvested using ice-cold RIPA Lysis and Extraction Buffer (Thermo Fisher Scientific, 89900) supplemented with 1X Halt^TM^ Protease Inhibitors Cocktail (Thermo Fisher Scientific, 78429). Total protein was quantified using the BCA assay (Thermo Fisher Scientific, 89900). 10 μg total protein was denatured at 100 °C for 5 min with 1X Laemlli and RIPA-supplemented protease inhibitor. Total protein was run on 4–12% Bis-Tris NuPage Gel (Invitrogen, NP0323BOX) under reducing conditions at 120 V for 90 min, protein was subsequently transferred to methanol-activated PVDF membrane at 75 V for 90 mins. Membranes were blocked in 5% (w/v) skim milk in Tris-buffered saline with Tween-20 (TBST), after which membranes were stained for either MECR (Thermo Fisher Scientific, PA5-54555, RRID:AB_2643839, 1:250), GATA4 (Abcam, ab124265, RRID:AB_11000793, 1:1000), ISL1 (Abcam, 86472, RRID:AB_1951287, 1:1000), NKX2.5 (Santa Cruz Biotechnology, sc-14033, RRID:AB_650281, 1:250), MYH6 (Abcam, ab50967, RRID:AB_942084, 1:1000), SLC8A1 (Cell Signaling, 55075-1-AP, RRID:AB_2881262, 1:1000), ACTN2 (Sigma, A7811, RRID:AB_476766, 1:1000), ACTC1 (Sigma, SAB5600071, 1:1000), ANKRD1/CARP (Millipore, MABS1228, 1:250), CRHBP (Sigma, HPA046120, RRID:AB_10959760, 1:1000), TNNT2 (Abcam, ab91605, RRID:AB_2050427, 1:5000), IGFBP7 (R&D systems, AF1334, RRID:AB_2264436, 1:200), CCDC80 (R&D Systems, AF3410, 1:1000) TNNI1 (Sigma, AV42117, RRID:AB_1858352, 1:1000), MYL4 (Abcam, ab231800, 1:1000) or actin (Millipore, MAB1501R, RRID:AB_2223041, 1:10000). Membranes were further washed with TBST and incubated with horseradish peroxidase-conjugated secondary antibodies in 5% (w/v) skim milk in TBST. Membranes were finally washed and developed using Amersham ECL Prime Western Blotting Detection Reagent (Cytiva, RPN2232) and detection was performed using a ChemiDoc MP Imaging System (Bio-Rad). All blots derive from the same run and they were processed in parallel. Densitometry was performed using ImageJ and normalized to actin. Data represent the average of 5 independent biological replicates from different differentiation batches. Supplementary Fig. 5 shows the raw blot images.

### Optical electrophysiology for detecting membrane electrical activity and data analysis

Day 9 CCPs were seeded onto custom-built 30-mm glass bottom dishes pre-coated with LN-521 and cell culture was maintained in cardiomyocyte maintenance medium (RPMI-1640 with 2% B27 supplement (Thermo Fisher Scientific, USA) with media exchanged every 2 days. Cultures were recorded at 11 and 17 days of differentiation as previously described^2^. Briefly, cell cultures were incubated with a voltage-sensitive dye, FluoVolt (Thermo Fisher Scientific, USA), and emitted absolute fluorescence signals captured on a custom-built high throughput OptioQUANTTM imaging platform (Ternion Biosciences, Singapore). All optical recordings were performed at physiological temperatures (35–37 °C). All data were analyzed with Image J and Igor Pro (WaveMetrics, USA)., and values were given as mean ± SEM. We applied classical wave analyses with triangulation, APD30, APD50, APD80, and APD90^3–6^ to characterize these regions and obtained these three representative traces. Analysis of these traces suggested classification as Ventricular-like, Atrial-like, and Nodal-like action potential waveforms at the time point.

### Teratoma formation assay

A teratoma formation assay in nude mice (8–10 weeks old, 20–25 g) was performed to investigate the in vivo safety of the cells. Single suspension of pluripotent H1 or day 11 CCPs (5 million cells) were resuspended in 100 μl of Matrigel^TM^ (Corning) and injected intramuscularly into the hind limb muscle for 8 weeks. The viability of the cells was determined by IVIS imaging by first intraperitoneally injecting D-luciferin (15 mg/ml in 100 μl) (Perkin Elmer, 122799-5) and then imaging the mice.

### Pig model

Either gender of three-month-old Sus scrofa pigs weighing 13–15 kg that were housed individually was purchased from SingHealth Experimental Medicine Centre (SEMC, Singapore). Pigs were allocated into the CCP-transplanted or medium-control group by randomizing the order of treatments and cardiac functional measurements into different surgery days. The animal surgeon, veterinarians, cardiac imaging specialists, electrophysiologists, and cardiac clinicians were unaware of the group allocation and treatment type. The personnel who were aware of the group allocations are the cell biologists who prepared the cells. Data were also analyzed in a blinded fashion by the clinicians and the outcome of the assessment was statistically determined.

The pigs were immunosuppressed to prevent rejection of the human cells in their heart. Five days before surgery, cyclosporine (Novartis, ADP835296) was given in the diet twice daily at 15 mg/kg and maintained throughout the experiment. The first dose of abatacept (Orencia®, Bristol-Myers Squibb, ABT4318) was given on the day of surgery at 12.5 mg/kg via intravenous injection and once every 2 weeks until the end of the experiment. Corticosteroid immunosuppression using methylprednisolone (*Vem ìlaç*, 011001) was administered intraperitoneally once on the day of surgery at 250 mg, followed by 125 mg for the first 2 weeks after transplant and, thereafter, 62.5 mg until the end of the experiment.

On the first 14 days post-surgery, antibiotics (Betamox (every other day) and Baytril (once daily)) were administrated to prevent infections. Analgesic (buprenorphine (twice daily)) was also administrated on the day of surgery and after electrophysiology mapping. To overcome the side effects of immunosuppression, iron capsules (26 mg, Now Foods) were given through diet every other day and ranitidine (twice daily) to prevent stomach ulcers.

Baseline heart function was determined in 10 pigs using MRI before any intervention (pre-MI) was given. The sample size was decided based on previous publications^5,37^ and statistical analysis was performed. On the day of surgery, the pigs were randomly assigned to two groups: the first group was injected with medium only (4 weeks, *n* = 4, 12 weeks, *n* = 6) and the second group was transplanted with day 11 CCPs (4 weeks, *n* = 3, 12 weeks, *n* = 7). Telazol (0.06–0.08 ml/kg, intramuscular injection (i.m.) was given for anesthesia induction, followed by general surgical anesthesia (ketamine 100 mg/kg + xylazine 20 mg/kg, i.m. and isoflurane 0.5–2.5%, inhaled). During surgery, adequate anesthesia was judged by the loss of muscle tone in the jaw and limbs, loss of corneal reflex, and absence of spontaneous respiration. Heart rate, respiratory rate, and temperature were monitored every 30 min. Post-operative analgesia and antibiotics were used at day 0–3 post-surgery: Ketoprofen 5 mg/kg/day, Enrofloxacin (Baytril: 15 mg/kg). The pigs were physically monitored by the veterinarians daily and blood biochemistry tests were performed every 2 weeks to detect any abnormal changes. At any given time during the research study, animals that suffer from severe or chronic pain and distress that cannot be relieved with the therapeutic intervention were painlessly euthanized. Examples of criteria used for euthanization are inability to eat or drink, signs of toxicity, and severe bleeding in the animals.

MI was generated by permanent ligation of the first branch of the left anterior descending coronary (LAD) and left circumflex (LCX) arteries, followed by intramyocardial injection of ~200 million luciferase-labeled CCPs in 1 ml of RMPI + B27 (without insulin medium) at around 10 sites (100 μl/site) into the peri-infarcted and infarcted region^9^. Pluripotent stem cells (H1) were transduced with lentivirus carrying the plasmid for luciferase-GFP-puromycin construct according to our previously published paper^1^. To ensure the luciferase-labeled CCPs are genetically stable after the transduction, karyotype analysis was performed on the stable cell line. For euthanization, pigs were first induced with ketamine/xylazine (ketamine 100 mg/Kg, xylazine 10 mg/kg, i.m.) after which potassium chloride (KCl, 100 mg/kg) was directly injected into the pig’s heart to ensure that the heart stops at the diastolic stage.

### Generation of myocardial infarction model and CCP transplantation

To track the biodistribution and viability of the progenitors, we employed our previously generated luciferase-labeled H1 cells in the pig experiments^1^. Briefly, luciferase-labeled CCPs were generated with replication incompetent RediFect Red-Fluc-Puro lentiviral particles (Perkin Elmer, Cat.#CLS960002) using 10 MOI in 10,000 pluripotent H1 cells. Positive clones were selected with 200 ng/ml of puromycin and assayed with D-luciferin (Perkin Elmer, Cat.# 760505) in Infinite 200 PRO series microplate reader (Tecan) at 1 sec integration time.

The pigs were anesthetized, intubated, and maintained through a ventilator. A limited left lateral thoracotomy was performed to expose the heart. MI was generated by permanent ligation with a nonabsorbable suture (B Braun, C0026003) of the first branch of the left anterior descending coronary (LAD) and left circumflex (LCX) arteries, followed by intramyocardial injection of 200 million luciferase-labeled CCPs into the infarcted region. This consistently gave a scar size of approximately 15% of the left ventricular anterior wall^10^. In the treated group, single-cell CCPs (200 million cells) in RMPI medium at 1 ml were intramyocardially injected into infarcted and peri-infarcted myocardium at several points (~10 sites). For the medium control pigs, 1 ml of RPMI medium was similarly intramyocardially injected into the infarcted region. The chest was closed-up and the animal was monitored closely after the surgery. An implantable loop recorder was implanted into the chest wall for daily ECG recording and transmission to the Reveal LINQ^TM^ cardiac monitoring system (Medtronic).

Animals were maintained for 4 or 12 weeks. A schematic representation of the surgery procedure is shown in Fig. 3a.For euthanization, pigs were first induced with ketamine/xylazine (ketamine 100 mg/Kg, xylazine 10 mg/kg, i.m.) after which potassium chloride (KCl, 100 mg/kg) was directly injected into the pig’s heart to ensure that the heart stops at the diastolic stage.

### Bioluminescent measurements

After euthanizing the pig, D-luciferin (15 mg/ml in 2 ml volume) (Perkin Elmer, 122799-5) was administered into the excised whole heart via injection into both coronary arteries. Ten minutes after the injection of D-luciferin, the right ventricle was removed and the left ventricle was cross-sectioned into 5 rings. All 5 rings were placed on the IVIS Spectrum imaging platform (Perkin Elmer) and 2D bioluminescent images were taken to calculate bioluminescent signals and total photons emitted from the heart area. Areas on the tissue rings with positive signals were sectioned and fixed in 4% PFA (Sigma, 28908) for downstream tissue processing and immunostaining.

### Immunohistology staining

Pig hearts were coronally dissected and transversely sectioned into five slices (15 mm thick) at 4 or 12 weeks post-MI. The infarcted area and border zone tissues were further dissected and fixed in 10% neutral buffered formalin (Sigma, HT501128) overnight at room temperature, processed, and paraffin-embedded for histological analyses. Tissues were sectioned into 5 μm thick sections followed by de-paraffinization and heat-induced antigen retrieval using sodium citrate buffer, pH 6 (Sigma, C9999) for 10 mins. After which, sections were washed thrice with 1 X PBS for 5 mins each and incubated with goat serum (Sigma, G9023) and 0.2% Triton X-100 (Sigma, T8787) for 30 mins. Sections were then incubated with specific primary antibody: anti-TNNT2 (Abcam, ab91605, RRID:AB_2050427, 1:100), anti-Ku80 (Cell Signaling, 2180 S, RRID:AB_2218736, 1:300), anti-Ku80 conjugated 488 (Abcam, ab198586, 1:100), anti-MLC2v (Abcam, ab79935, RRID:AB_1952220, 1:100), anti-ACTN2 (Sigma, A2172, RRID:AB_476695 1:500), anti-N-cadherin (Sigma, C3678, RRID:AB_258851, 1:100), anti-CX43 (Sigma, 6219 C, 1:200), anti-CD31 (Abcam, ab28364, RRID:AB_726362 1:100), anti-TNNI3 (Novus, NBP1-56641, RRID:AB_11035917, 1:100), anti-MLC2a (Sigma, HPA013331, RRID:AB_1854245, 1:100), anti-Ki67 (Abcam, ab15580, RRID:AB_443209, 1:100), anti-PPH3 (Cell Signaling, 9701, RRID:AB_331535, 1:100), anti-CD45 (Bio-Rad, MCA1447, RRID:AB_2174248, 1:100), anti-CD20 (Biocare Medical, ACR3004B, 1:100) and anti-CD3 (DAKO, A0452, RRID:AB_2335677 1:100) 4 °C overnight. The next day, sections were washed thrice with 1 X PBS for 5 mins each. Alexa-conjugated secondary antibody (ThermoFisher, 1:1000) and DAPI (ThermoFisher, D1306, RRID:AB_2629482 1:5000) were added for 1 hr and washed thrice with 1 X PBS for 5 min each. To suppress autofluorescence, the sections were incubated with Sudan Black (Sigma, 199664) for 20 mins, washed thrice with 1 X PBS, and then mounted with ProLong Gold antifade mountant (ThermoFisher, P36930). Slides were examined using LSM 710 Carl Zeiss confocal microscope^11^.

Infarct size, blood vessel density, and gap junction were determined from images stained with ACTN2, CD31, and Cx43 antibodies respectively. Images from 3 different areas (remote, infracted, and graft) were obtained using 10x magnification from 3 different areas in 3 pigs each. Stains were quantified using the Fiji image processing package and calculated by (number of spots)/ positive area (mm^2^).

The proliferation rate of the engrafted cardiomyocytes was determined from sections stained for Ki67 and PPH3. Analysis was done blindly by manually counting the Ki67+ and PPH3+ nuclei from the average of five fields per heart (40 X objective) by a researcher. The proliferation rate was expressed as % of proliferation = (the number of Ki67 + /PPH3 + ) divided by (the number of Ku80+ human nuclei).

### Hematoxylin and eosin staining

Sections were deparaffinized in 2 changes of Histoclear (Nanoserv, 64110-04), 2 mins each, followed by 100% ethanol and 70% ethanol, washing in water for 5 mins. After rehydration, sections were incubated in hematoxylin (Biomed Diagnostics, 3801520) for 10 mins, washed thrice in water for 5 min each followed by 1 min incubation in Eosin (Leica, 3801601). For dehydration, slides went through a series of changes of ethanol (70% to 100% ethanol), and 3 changes of Histoclear, and were mounted with mounting media (Leica, 1407093626). Slides were examined using a Leica DMi8 microscope.

### Masson trichrome staining

Deparaffinized and rehydrated sections were fixed in Bouin’s (Sigma, HT10132) solution for 1 hr at 60 °C. Rinsed sections in running tap water for 15 min to remove the yellow color. Sections were stained in Weigert’s Iron Haematoxylin solution (Merk, 1.15973.0002) for 10 min, washed in water for 5 mins, and stained in Biebrich Scarlet-Acid Fuchsin solution (Sigma, B6008 and F8125) for 3 min and rinsed with water. After that, differentiate in phosphomolybdic-phosphotungstic acid solution (Nanoserv, 19400 and 19500) for 10 min, transferred sections directly to methylene blue solution (Merk, 1.15943.0025), and stained for 10 mins. Rinsed briefly in water and differentiated in 1% acetic acid solution (Sigma, 695092) for 5 mins. Dehydrated and mounted the slides as previously mentioned. Examined the slides using Leica DMi8 microscope. Stained sections were analyzed by Fiji image processing package (version 2.3.0/1.53t) to quantify the percentages of fibrosis in each tissue.

### Cardiac MRI scan and analysis

Cardiac Magnetic resonance imaging (MRI) was performed at 1, 4, and 12 weeks (for pigs with 12-week follow-up) post-surgery. Replicates number for week 1 (*n* = 10 medium control, *n* = 10 CCP transplanted), week 4 (*n* = 10 medium control, *n* = 10 CCP transplanted) and week 12 (*n* = 3 medium control, *n* = 5 CCP transplanted) weeks post cell-transplantation (refer to Table 1). The scans were conducted on a 3.0 T whole-body MRI machine (Siemens Skyra, Siemens Medical Systems, Erlangen, Germany) with a standard cardiac flex coil (Siemens Medical Systems, Erlangen, Germany) as described^10,12^. Briefly, the scan protocol consisted of: 1) localizing scouts to identify the long- and short-axis of the heart, 2) short- and long-axis cine (Steady-state free precession “True-FISP” sequence) for the measurement of global cardiac function, and 3) delayed contrast-enhancement imaging (turbo-FLASH sequence) for the assessment of scar size. Image acquisition will be conducted with both electrocardiogram (ECG) and respiration gating. For the late-gadolinium imaging were done 8 mins after dotarem injection, an appropriate inversion time (TI) will be chosen to adequately null the signal intensity (SI) of normal myocardium. Infarct size will be calculated from the delayed contrast-enhanced images to manually segment regions of non-viable tissue.

MRI imaging was performed by staff who were blinded to the animal groups. Pigs were sedated with intramuscular injection of ketamine/xylazine mixture. Anesthesia was maintained with 2–2.5% isoflurane on a ventilator after intubation. Physiological monitoring will be applied and recorded. Such as heart rate, sPO2, body temperature monitored by thermal catheter, heating fan, CO2%, IV access for 0.9% NaCl dripping, and injecting contrast. If the heart rate is low, atropine (0.04 mg/kg, i.v.) will be administered by the vets in NUS CM. The animals will be mechanically ventilated during scans, and pancuronium (0.1 mg/kg, i.v.) may be given to facilitate breath holding. The vital signs such as heart rate, respiration rate, and body temperature will be continuously monitored while the animal is anesthetized. The animal is also mechanically ventilated during the scans. LV function was investigated using a segmented breath-held steady-state free precession cine MRI imaging sequence. Contiguous 10 to 12 short-axis 2D slices without any gap covering the LV from base to apex were acquired.

Global function (Left ventricular ejection fraction (LVEF)) was computed from the short-axis cine images by semi-automated segmentation of the LV endocardial and epicardial borders (from base to apex) at both end-diastole and end-systole using CVi42 analysis software (Circle Cardiovascular Imaging Inc., Canada)^10^.

Infarct size was calculated from the late gadolinium enhancement images with scar surface area expressed as a percentage of the total LV surface area^10^. Briefly, within the defined endocardial and epicardial borders, the LV area with signal intensity > mean+2 SD of non-infarcted septal wall intensity was considered a scar. Wall thickness (mm) was also measured from the software and the results were represented with AHA segmentations in a polar map by registering the endocardial and epicardial contours of corresponding end-systolic and end-diastolic images. Since the segment of interest that is affected by the infarction is segment 16, and in order not to lose the sensitivity of the data from a small region, we will only compare the values from this segment.

### Computerized tomography (CT) scan

Whole-body CT scans were acquired using a dual source 256 slices Siemens Somatom Definition Flash-CT camera. Animals were anesthetized, intubated, maintained on isoflurane, and kept warm throughout the imaging procedure. Ketamine (100 mg/kg + xylazine 20 mg/kg, i.m.) was used for induction and 2–3% isoflurane was used for maintenance. Physiological monitoring was done on heart rate, sPO_2_, and CO_2_ %. The animals were kept warm by a convection warming blower, body temperature monitoring was done using a rectal probe. There was IV access for 0.9% NaCl drip and injecting contrast.

CT scans were done with the animal placed in the supine position. An intravenous catheter was inserted into the ear vein and connected to a contrast injector. The scan area included the pig’s snout superiorly and the lower limb inferiorly. There were 4 scan phases, pre-contrast, arterial phase (cardiac scan only), portal venous, and delayed phase. Whole body scans were done in inspiration breath hold while scans over the chest were additionally ECG-gated. The contrast agent (Omnipaque 350®) was given at 1–3 ml/kg together with a saline bolus flush at 20 ml or 0.5 ml/Kg of the pig’s weight depending on which was lower. Beta-blockers (Esmolol 0.4 mg/kg) were on hand to be used if the pig’s heart rate went above 75 bpm to ensure the highest resolution of the cardiac images. Images were reconstructed and viewed on a SyngoVia workstation and analyzed to identify tumor formation in any organs. If a tumor was found, a necropsy will be performed when animals are sacrificed. The tumor will be explanted, fixed, and sectioned to determine whether it was a teratoma or other tumor original from human cells by performing PCR for human Y chromosome detection.

### Electrophysiology analysis

Electromechanical mapping using the 3D-NOGA system simultaneously registers the electrical and mechanical activities of the left ventricle, enabling the assessment of myocardial viability. The aim of NOGA was to identify and localize arrhythmogenic foci and direct therapeutic procedures. This 3D mapping was done at 2 and 4 weeks by the electrophysiologist (EP) mapper and the technical support team from Johnson and Johnson.

The external reference patch was placed on the pig’s back, behind the heart. A 7 French introducer sheath was inserted into the right carotid artery, followed by the administration of 0.9% NaCl/saline containing 1,000 IU of heparin. The fully deflected NOGA-Star (Biosense Webster) catheter was advanced to the ascending aorta. The NOGA-Star catheter was moved through the aortic valve and the catheter tip was oriented toward the apex. An apical point was obtained, followed by an outflow tract, lateral, and posterior points, to form a 3D silhouette of the heart. The catheter was then deflected toward the anterior wall of the ventricle, during which multiple points of the anterior area were acquired with the homogeneous distribution. The process was repeated to obtain additional reference points from the lateral, septal, anterior, and inferior walls, thus obtaining a full 3D map of the left ventricle.

During the mapping procedure, the NOGA workstation analyzed the 3D location of the catheter tip on the endocardial surface. The catheter tip moved in a stepwise manner to gather simultaneous unipolar and bipolar electrical signals for each mapping point. The stability of the catheter-to-wall contact was evaluated at every site in real-time. After completing the map, postprocessing analysis filtered the unstable points caused by rhythm disturbances or internal cavities in the left ventricle. For all pigs in sinus rhythm, sinus rhythm left ventricular activation time mapping and voltage mapping were performed. For pigs with sustained ventricular tachycardia (VT), the VT was similarly mapped. VT was characterized as focal if there was a radial spread of electrical activation from a point source.

Image and video postprocessing was performed with assistance by the team from Johnson and Johnson in a blind fashion. Unipolar voltage maps indicate the exact positions of infarction. Unipolar voltages lower than 5 mV are considered scar (red color), and unipolar voltages 5 mV and more are considered healthy and viable myocardium (purple color). A local linear shortening (LLS) map provides information about wall movement (red = low movement, purple = healthy movement).

### Implantable cardiac monitoring system

During the surgical procedure, the Reveal LINQ^TM^ cardiac monitoring system (Medtronic, USA) was subcutaneously placed in the left paraspinal area inferior to the angle of the scapula in pigs after surgery. The minimum sensed R wave post-fixation of the Reveal LINQ was at least 0.3 mV. Programmed settings were: sensitivity- 0.035 mV, Blank-after-sense 150 ms, sensing-threshold decay 150 ms, ectopy rejection off. Recordings were intermittently downloaded once every 2 days. Detection criteria used were: Tachycardia – cycle length ≤370 ms (162 bpm), number of intervals to detect (NID) 48 beats; Bradycardia – cycle length ≥2000 ms (30 bpm), NID 4 beats; Pause – cycle length ≥3000 ms. Every download was classified into three categories: (1) ventricular arrhythmia (defined as runs of 4 or more QRS complexes of different morphology compared to baseline or temporally adjacent sinus QRS together with evidence of atrioventricular dissociation), (2) bradycardia (defined as evidence of high-grade atrioventricular block, sinus bradycardia ≤30 beats per minute and/or 3 or more sinus pauses or arrest with or without junctional escape rhythm within one recording), or (3) normal (downloads not belonging to the other two categories). However, there are limitations in this loop recorder system such as the detection of arrhythmia which is based on rate thus slow VT may not be recorded and pigs intrinsically have long QT intervals, so that T (and also even P) wave oversensing often occurs. Due to frequent T wave oversensing and QRS under sensing, all recordings were individually and manually read by an experienced electrophysiologist (Dr. Eric Lim, NHCS). Equivocal recordings were adjudicated by a second electrophysiologist (Dr. Paul Lim, NHCS).

### Statistical analysis

Data were also analyzed in a blinded fashion by the clinicians and the outcome of the assessment was statistically determined. Pigs with heart rates that were too fast to be imaged by MRI will be excluded from the analysis. Comparisons between groups were performed using two-way ANOVA with Tukey post hoc analysis unless otherwise stated. Values were reported as Mean ± SEM. Data were analyzed by GraphPad Prism (Version 9). Replicate information is indicated in the figure legends and methods. A *p*-value < 0.05 was considered statistically significant. *p < 0.05, **p < 0.005 and ***p < 0.0005.

## Supplementary information

Supplementary information

nr-reporting-summary

Supplementary Video 1

Supplementary Video 2

Supplementary Video 3

## Data Availability

The bulk and single-cell RNA sequencing data is made available on Gene Expression Omnibus with accession number GSE204881. The non-sequencing data and materials are available from the corresponding author upon reasonable request.
