# Supplementary material for: Pluripotent stem cell-derived committed cardiac progenitors remuscularize damaged ischemic hearts and improve their function in pigs
Source: NPJ Regen Med. 2023 May 26;8:26. doi: 10.1038/s41536-023-00302-6 (PMC10219927; doi:10.1038/s41536-023-00302-6)
Supplement: Supplementary file 1 — Supplementary information [file 41536_2023_302_MOESM1_ESM.pdf]

Pluripotent stem cell-derived committed cardiac progenitors remuscularized and improve the heart function of ischemic pig hearts

Lynn Yap[1,2*]; Li Yen Chong[1]; Clarissa Tan[1]; Swarnaseetha Adusumalli[1]; Millie Seow[1]; Jing Guo[1]; Zuhua Cai[1]; Sze Jie Loo[3]; Eric Lim[3]; Ru San Tan[3]; Elina Grishina[4]; Poh Loong Soong[4, 5]; Narayan Lath[3]; Lei Ye[6]; Enrico Petretto[1]; Karl Tryggvason[1, 7, 8*]

[1]Cardiovascular & Metabolic Disorders Program, Duke-NUS Medical School, National University of Singapore, Singapore 169857, Singapore

[2]Lee Kong Chian School of Medicine, Nanyang Technological University, 11 Mandalay Road, Singapore 308232, Singapore

[3]National Heart Research Institute Singapore, National Heart Centre Singapore, Singapore, 169609, Singapore

[4]Ternion Biosciences, Singapore 574329, Singapore

[5]Cardiovascular Disease Translational Research Program, Yong Loo Lin School of Medicine, NUS, Singapore 169609, Singapore

[6]Department of Biomedical Engineering, University of Alabama, Birmingham, 35233

[7]Department of Medicine Duke University, Durham, North Carolina, 27710, USA

[8]Department of Medical Biochemistry and Biophysics, Karolinska Institute, 171 77 Stockholm, Sweden

* Co-corresponding Author

Supplementary Files

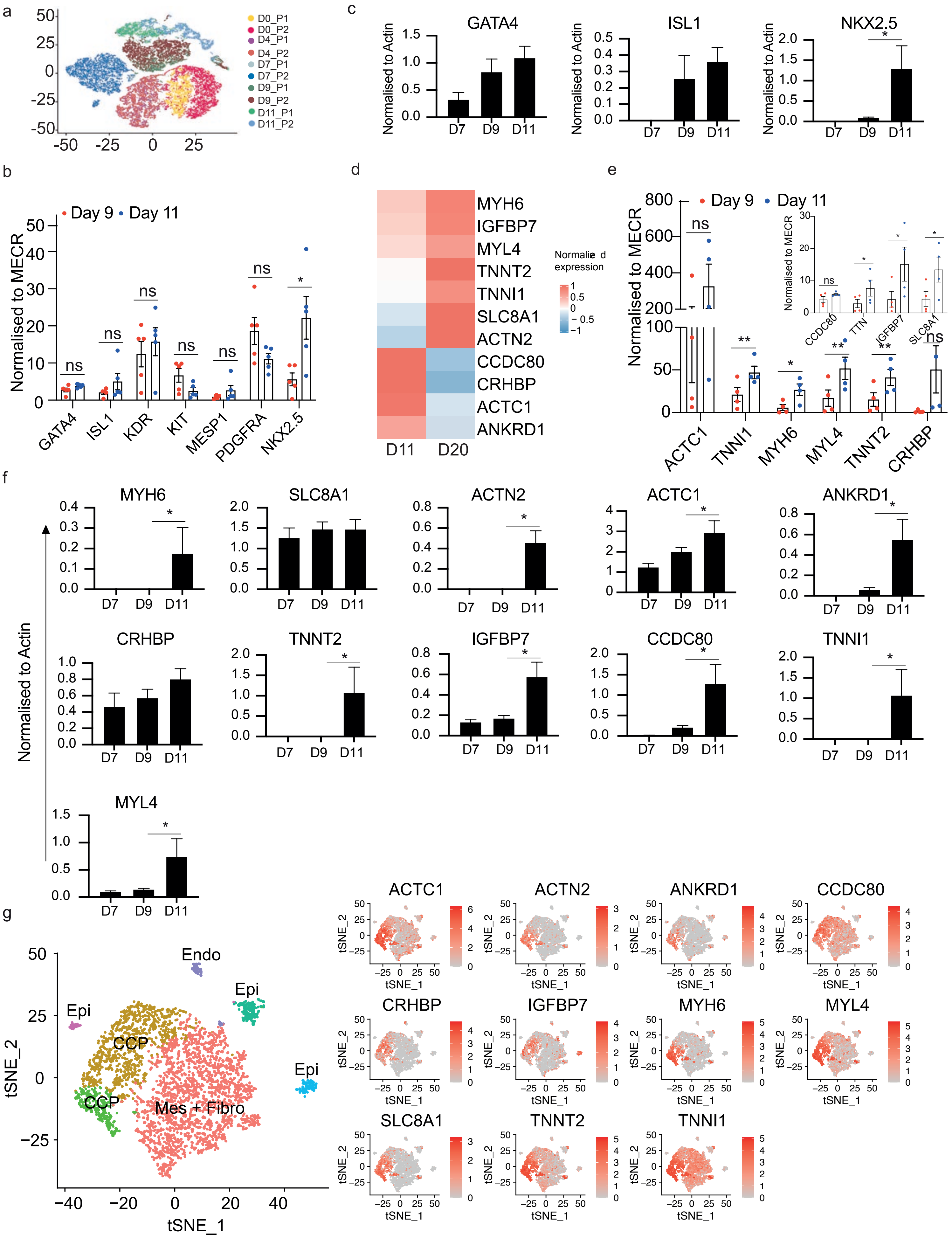
a
D0_P1
D0_P2
D4_P1
D4_P2
D7_P1
D7_P2
D9_P1
D9_P2
D11_P1
D11_P2
b
Day 9 Day 11
Normalised to MECR
GATA4
ISL1
KDR
KIT
MESP1
PDGFRA
NKX2.5
c
GATA4
ISL1
NKX2.5
Normalised to Actin
D7 D9 D11
d
MYH6
IGFBP7
MYL4
TNNT2
TNNI1
SLC8A1
ACTN2
CCDC80
CRHBP
ACTC1
ANKRD1
D11 D20
e
Day 9 Day 11
Normalised to MECR
ACTC1
TNNI1
MYH6
MYL4
TNNT2
CRHBP
CCDC80
TTN
IGFBP7
SLC8A1
f
Normalised to Actin
MYH6
SLC8A1
ACTN2
ACTC1
ANKRD1
CRHBP
TNNT2
IGFBP7
CCDC80
TNNI1
MYL4
g
Endo
Epi
CCP
Mes + Fibro
tSNE_1
tSNE_2

Supplementary Figure 1. Characterization of CCPs.

(a) The tSNE plot of H1 single-cell RNAseq data from 2 biological replicates on days 0, 4, 7, 9, and 11. It shows the highly reproducible overlaps from both passages each day. (b) Quantitative PCR of canonical progenitor genes from differentiation days 9 and 11. The genes were normalized to the expression of the MECR gene. There are no significant differences between days 9 and 11 for all the genes (p-value > 0.05) except NKX2.5 (* p-value < 0.05). Mean ± SEM, n=6 individual differentiation batches. (c) Densitometry analysis of immunoblots of canonical progenitors markers in Figure 1e. p-value < 0.05, n = 5**.** (d) Heatmap of CCP genes on days 11 and 20. (e) Quantitative PCR results of day 11 CCP signature genes from differentiation days 9 and 11 (four biological replicates). The genes are normalized to MECR. Significant differences on day 11 are observed in all the genes (p-value < 0.05) except for ACTC1 and CCDC80 (* p-value < 0.05, ** p-value < 0.005) when compared with day 9. Mean ± SEM, n=6 individual differentiation batches. (f) Densitometry analysis of immunoblots CCP signature genes in Figure 1i. p-value < 0.05, n = 5 (g) The tSNE plot of the scRNA-seq data displays the subpopulations of CCP, mesenchymal + fibroblasts (Mes + Fibro), epithelial (Epi), and endothelial (Endo) on day 11. Individual genes are plotted on the right.

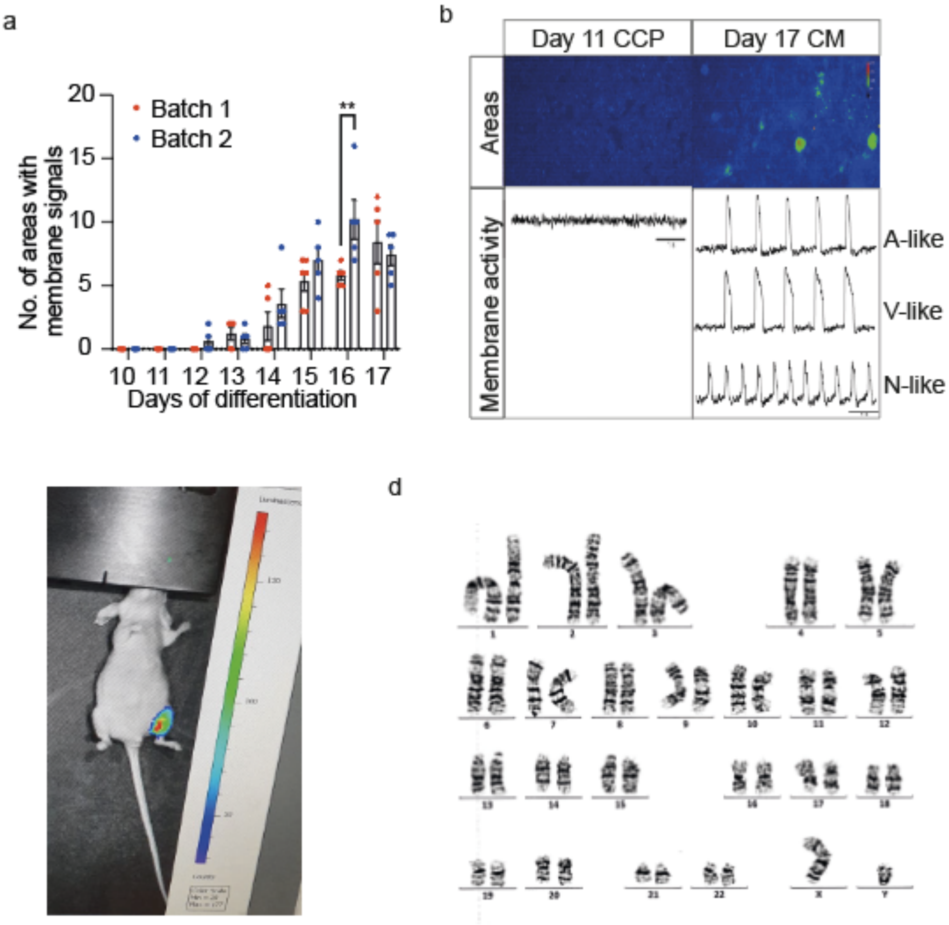

Supplementary Figure 2. Characterization of CCPs

(a) Optical electrophysiology results showing the number of areas with membrane potential across days of differentiation from 2 batches of differentiation (p-value < 0.05). (b) Representative image of day 11 CCP and 17 days of differentiation with detectable membrane potential (green spots). Action potential traces at 11 days of differentiation showed the absence of signals while membrane activities of atrial-like (A), ventricular-like (V), and nodal-like (N) CM were observed on Day 17. (c) IVIS optical scan image of CCP-transplanted nude mice showing the location of luciferase-positive CCP after 8 weeks and the absence of abnormal tumor growth at the hindlimb. (d) A normal karyogram of luciferase-transduced H1 cells.

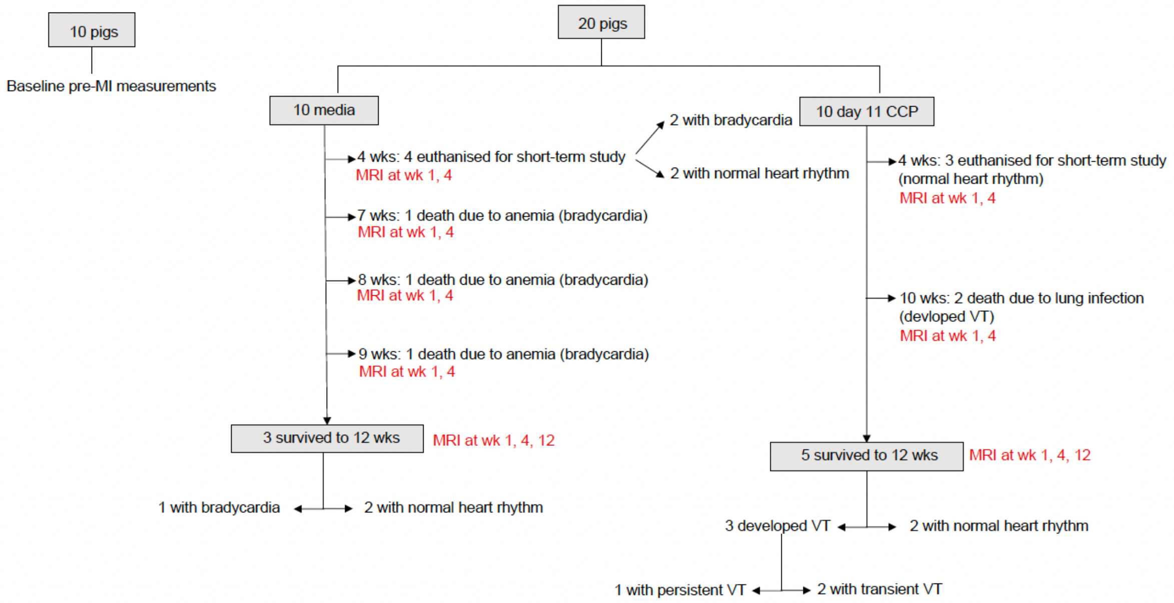

Supplementary Figure 3. Flowchart to describe the overall experimental design of the study

A total of 20 pigs were enrolled in the study. 10 pigs for media control and 10 pigs for CCP transplantation. An independent 10 pigs for pre-MI baseline measurements.

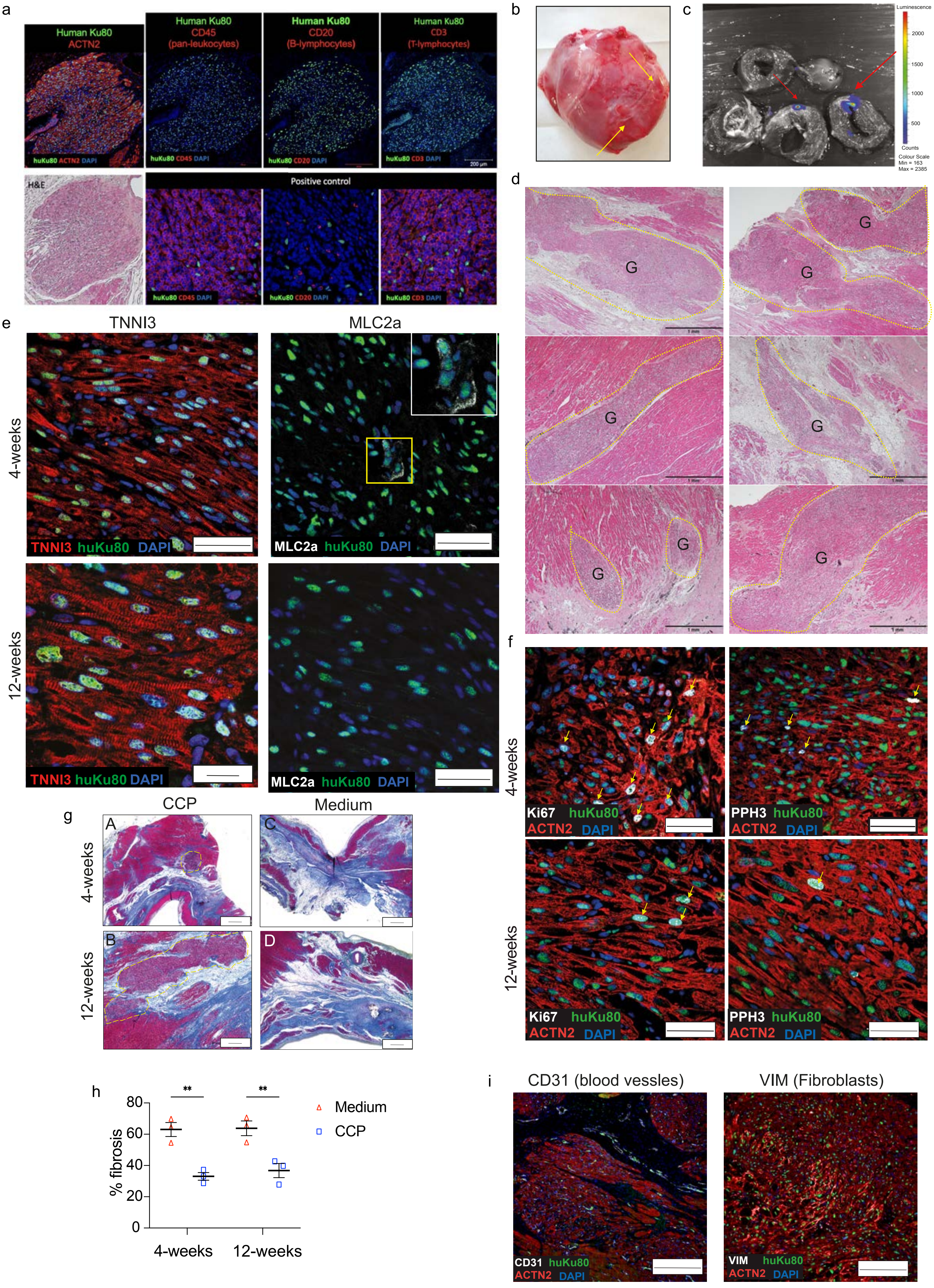
a
Human Ku80
ACTN2
Human Ku80
CD45
(pan-leukocytes)
Human Ku80
CD20
(B-lymphocytes)
Human Ku80
CD3
(T-lymphocytes)
H&E
Positive control
b
c
Luminescence
Counts
d
G
e
TNNI3
MLC2a
4-weeks
12-weeks
TNNI3 huKu80 DAPI
MLC2a huKu80 DAPI
f
Ki67 huKu80
ACTN2 DAPI
PPH3 huKu80
ACTN2 DAPI
g
CCP
Medium
A
B
C
D
h
% fibrosis
Medium
CCP
4-weeks
12-weeks
i
CD31 (blood vessels)
VIM (Fibroblasts)
CD31 huKu80
ACTN2 DAPI
VIM huKu80
ACTN2 DAPI

Supplementary Figure 4. Characterization of human grafts at 4- and 12-weeks. (a) Top: Confocal image of human graft stained with huKu80 (green) and ACTN2 (red) indicating the presence of human cells in the graft. Confocal images of human grafts stained with immune cell markers in red CD45 (pan-leukocytes), CD20 (B-lymphocytes), and CD3 (T-lymphocytes), show the absence of these immune cells. Bottom: A representative H&E image of human graft in an infarcted area. Confocal images of tissues with immune cells as the positive control. Scale bar = 200 μm (b) Whole heart image demonstrating the presence of the ligation suture (yellow arrows) at the LAD artery after 12 weeks post-transplantation confirming the permanent MI model. Scale bar = 1 mm (c) *In vivo* IVIS imaging of hearts after CCP transplantation shows the presence of live human cells (bioluminescence signal, red arrows) at 12 weeks post-transplantation. (d) H&E staining on 12 weeks human grafts. Human grafts (indicated as g) are demarcated in dotted yellow lines. Grafts sizes range from 1 mm to 10 mm. (e) Confocal images of human grafts stained with (left) TNNI3 (red), huKu80 (green), and (right) atrial-like CM marker, MLC2a (white), huKu80 (green) at 4 and 12 weeks post-transplantation, Scale bar = 50 μm. No atrial-like CMs were present in the infarcted area. (f) Confocal images of tissue stained with proliferation markers, Ki67 or PPH3 (white), huKu80 (green), ACTN2 (red) at 4- and 12 weeks. Scale bar = 50 μm (g) Bright-field images of CCP transplanted tissues stained with Masson trichrome at 4 and 12 weeks post-transplantation. Scale bar = 1 mm (h) Graph showing the quantification of the Masson trichrome staining in medium and CCP-transplanted heart at 4 and 12 weeks. ** p-value < 0.01 (i) Immunocytochemistry of human grafts with human nuclei-specific Ku80 in green, blood vessels-specific CD31 or fibroblast-specific vimentin (VIM) in white, cardiac-related gene (ACTN2) in red and nuclei in blue. Scale bar = 50 μm

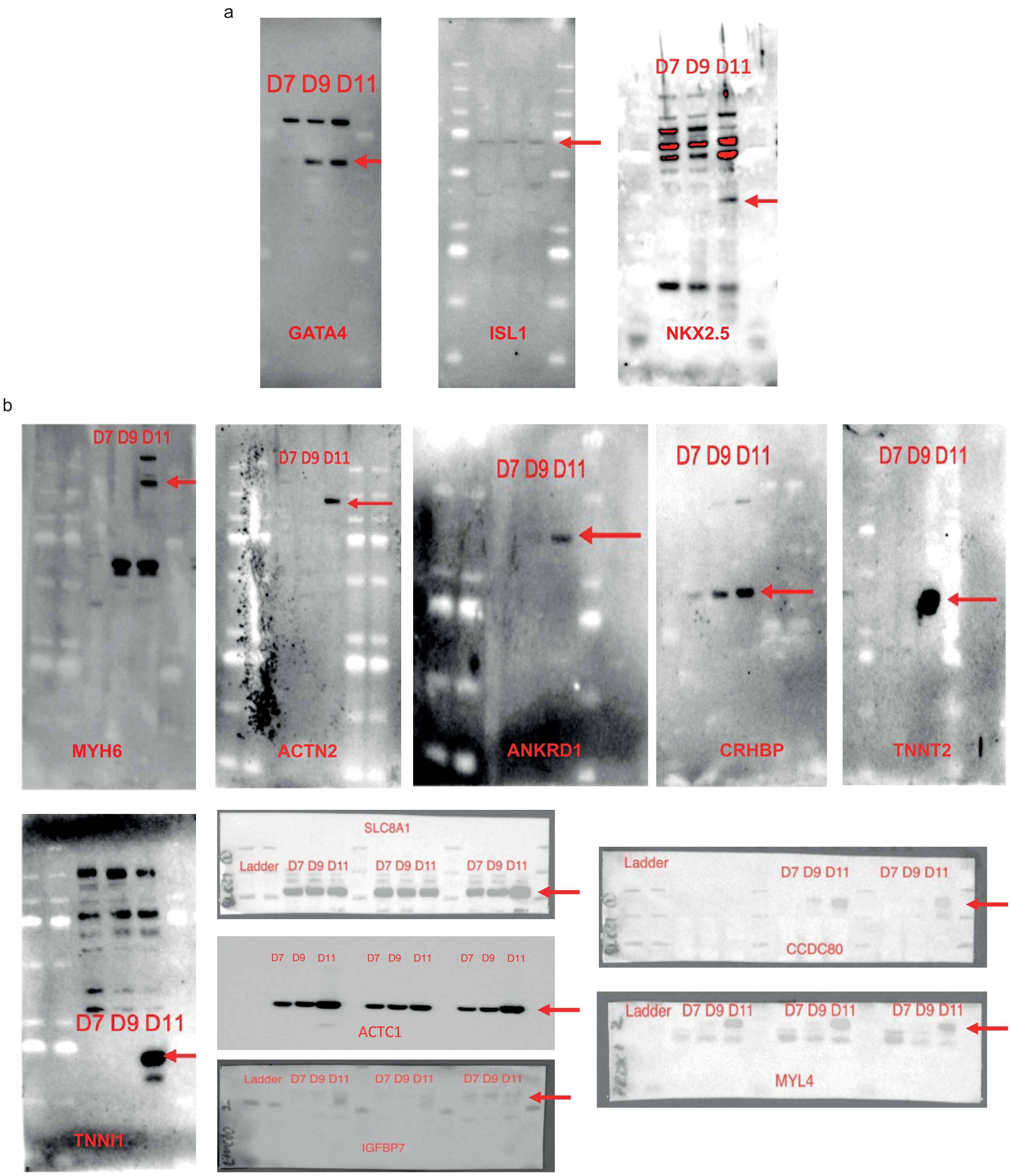
a
D7 D9 D11
GATA4
ISL1
D7 D9 D11
NKX2.5
b
D7 D9 D11
MYH6
D7 D9 D11
ACTN2
D7 D9 D11
ANKRD1
D7 D9 D11
CRHBP
D7 D9 D11
TNNT2
D7 D9 D11
TNNI1
SLC8A1
Ladder D7 D9 D11 D7 D9 D11 D7 D9 D11
D7 D9 D11 D7 D9 D11 D7 D9 D11
ACTC1
Ladder D7 D9 D11 D7 D9 D11 D7 D9 D11
IGFBP7
Ladder D7 D9 D11 D7 D9 D11
CCDC80
Ladder D7 D9 D11 D7 D9 D11 D7 D9 D11
MYL4

Supplementary Figure 5. Raw western blot images of marker genes

(a) Western blot images of GATA4, ISL1 and NKX2.5 (corresponding to Figure 1d)

(b) Western blot images of CCP signature genes including MYH6, SLC8A1, ACTN2, ACTC1, ANKRD1, CRHBP, TNNT2, IGFBP7, CCDC80, TNNI1 and MYL4. (corresponding to Figure 1h)

Supplementary Video 1: Video of medium control pig with activation mapping showing scarring in the myocardium with red color

Supplementary Video 2: Video of electromechanical mappings in CCP transplanted pig without VT.

Supplementary Video 3: Video of electromechanical mappings in CCP transplanted pig with VT.

Supplementary Table 1.

Bulk RNA sequencing fold change values between days 9 and 11 for the canonical and unique CCP signature genes.

Positive FC D9 > D11, Negative FC D11 > D9

	Row.names	baseMean	log2FoldChange	lfcSE	stat	pvalue	padj	significance
GATA4	ENSG00000136574	2389.01786	-0.092155466	0.33591788	-0.2616549	0.79358755	1	NS
ISL1	ENSG00000016082	476.805485	1.341505415	0.40814848	3.21672573	0.00129662	0.0583098	NS
KDR	ENSG00000128052	10333.1799	0.429613486	0.58713695	0.81272567	0.41637536	1	NS
KIT	ENSG00000157404	2062.1677	1.009000883	0.44049829	2.27789727	0.0227327	0.49207767	NS
MESP1	ENSG00000166823	36.6626216	1.463464972	0.57394073	2.54122217	0.01104657	0.30333907	NS
NKX2-5	ENSG00000183072	1237.02953	-3.655018313	0.49177469	-7.4007428	1.35E-13	1.22E-10	S
PDGFRA	ENSG00000134853	9888.13345	1.438401415	0.39158058	3.68803668	0.00022599	0.01358984	S

	Row.names	baseMean	log2FoldChange	lfcSE	stat	pvalue	padj	significance
MYH6	ENSG00000197616	80644.3594	-7.366190764	0.5943595	-12.232554	2.08E-34	3.16E-30	S
SLC8A1	ENSG00000183023	6487.12829	-5.262693064	0.55881536	-9.4151244	4.73E-21	1.44E-17	S
ACTN2	ENSG00000077522	4878.39653	-4.559286453	0.64020591	-7.14508	8.99E-13	6.65E-10	S
ACTC1	ENSG00000159251	19930.1004	-3.610740635	0.49608066	-7.2682712	3.64E-13	2.96E-10	S
ANKRD1	ENSG00000148677	6054.57918	-1.51859583	0.66290716	-2.2311825	0.02566904	0.5322672	NS
CRHBP	ENSG00000145708	834.902819	-6.123338416	0.49297893	-12.285963	1.08E-34	2.63E-30	S
TNNT2	ENSG00000118194	10767.1328	-4.25514647	0.56113053	-7.6264618	2.41E-14	2.45E-11	S
IGFBP7	ENSG00000163453	835.199649	-3.421883784	0.51601852	-6.6929085	2.19E-11	1.05E-08	S
CCDC80	ENSG00000091986	4297.85928	-3.527509781	0.48020768	-7.2968299	2.95E-13	2.57E-10	S
TNNI1	ENSG00000159173	6524.01948	-3.10845505	0.50272012	-6.1185238	9.44E-10	3.66E-07	S
MYL4	ENSG00000198336	6611.08959	-2.51896335	0.37625544	-6.6899396	2.23E-11	1.05E-08	S

Bulk RNA sequencing fold change values between days 11 and 20 for the canonical and unique CCP signature genes.

Positive FC D9 > D11, Negative FC D11 > D9

	Row.names	baseMean	log2FoldChange	lfcSE	stat	pvalue	padj	significance
MYH6	ENSG00000197616	80644.3594	0.394958942	0.59378669	0.74039518	0.45906024	0.71268542	NS
SLC8A1	ENSG00000183023	6487.12829	1.098161082	0.55667964	1.96789194	0.04908047	0.20149689	S
ACTN2	ENSG00000077522	4878.39653	1.504611393	0.63833236	2.3734839	0.01762116	0.10467334	S
ACTC1	ENSG00000159251	19930.1004	-1.254048375	0.49598309	-2.5053235	0.01223394	0.08277057	S
ANKRD1	ENSG00000148677	6054.57918	-0.820213048	0.66287653	-1.200178	0.23007023	0.49608664	NS
CRHBP	ENSG00000145708	834.902819	-4.697886262	0.48399967	-9.5674603	1.10E-21	1.48E-18	S
TNNT2	ENSG00000118194	10767.1328	0.534492526	0.5606345	0.97621133	0.32895975	0.60220516	NS
IGFBP7	ENSG00000163453	835.199649	0.25878806	0.51262256	0.51917518	0.60363859	0.8130151	NS
CCDC80	ENSG00000091986	4297.85928	-2.217962122	0.47992091	-4.6113509	4.00E-06	0.00016555	S
TNNI1	ENSG00000159173	6524.01948	0.508218711	0.50230891	1.04345645	0.29673694	0.57099093	NS
MYL4	ENSG00000198336	6611.08959	0.012536032	0.37596654	0.01951657	0.98442902	0.99458326	NS

Supplementary Table 2. Primer sequences

Num.	Primer ID	Primer sequence (5' → 3')
1	GATA4-F	GTT GCA CAG ATA GTG ACC CGT
2	GATA4-R	CGA CAC AAT CTC GAT ATG
3	ISL1-F	GAG GGT TTC TCC GGA TTT GG
4	ISL1-R	TCC CAT CC TAA CAA AGC ATG T
5	KDR-F	GGC CCA ATA ATC AGA GTG GCA
6	KDR-R	CCA GTG TCA TTT CCG ATC ACT TT
7	KIT-F	CGT TCT GCT CCT ACT GCT TCG
8	KIT-R	CCC ACG CGG ACT ATT AAG TCT
9	MESP1-F	TGT ACG CAG AAA CAG CAT CC
10	MESP1-R	TTG TCC CCT CCA CTC TTC AG
11	PDGFRA-F	TGG CAG TAC CCC ATG TCT GAA
12	PDGFRA-R	CCA AGA CCG TCA CAA AAA GGC
13	NKX2.5-F	ACC CTG AGT CCC CTG GAT TT
14	NKX2.5-R	TCA CTC ATT GCA CGC TGC AT
15	ACTC1-F	TCC CAT CGA GCA TGG TAT CAT
16	ACTC1-R	GGT ACG GCC AGA AGC ATA CA
17	TNNI-F	CCG GAA GTC GAG AGA AAA CCC
18	TNNI-R	TCA ATG TCG TAT CGC TCC TCA
19	MYH6-F	GCC CTT TGA CAT TGG CAC TG
20	MYH6-R	GGT TTC AGC AAT GAC CTT GCC
21	MYL4-F	ACT GCC GAC CAG ATT GAA GAG
22	MYL4-R	CTT GTT GCG GGA AAT GTG CTG
23	TNNT2-F	GGA GGA GTC CAA ACC AAA GCC
24	TNNT2-R	TCA AAG TCC ACT CTC TCT CCA TC
25	CRHBP-F	ATG TCG CCC AAC TTC AAA CTT
26	CRHBP-R	GCA GGA AAG GAT CGT AGT CCG
27	CCDC80-F	GCT GGT GAT CTC TGC TCC TAA C
28	CCDC80-R	CTT CCT CTC CAA CGC CTA AAA GC
29	TTN-F	CTG CTG ACT ACA CCT TTG TGG C
30	TTN-R	GCT CGC TTC TTC TCC AGT ACC T
31	IGFBP7-F	GCG AGC AAG GTC CTT CCA TA
32	IGFBP7-R	GGG ATT CCG ATG ACC TCA CA
33	SLC8A1-F	ACA ACA TGC GGC GAT TAA GTC
34	SLC8A1-R	GCT CTA GCA ATT TTG TCC CCA
35	MECR-F	CTG GCG GCC CCT ATC AAT C
36	MECR-R	AAC ACC TTC GTT CCC TCC AAC

Supplementary Table 3. Summary of MRI measurements

ID	Cause of death	1-week Heart rate (bpm)	1-week LVED V (ml)	1-week LVESV (ml)	1-week LVEF (%)	1-week Infarct size (%)	1-week Diastolic wall thickness (mm)	1-week Systolic wall thickness (mm)	1-week Wall motion (mm)	4-week Heart rate (bpm)	4-week LVEDV (ml)	4-week LVESV (ml)	4-week LVEF (%)	4-week Infarct size (%)	4-week Diastolic wall thickness (mm)	4-week Systolic wall thickness (mm)	4-week Wall motion (mm)	12-week Heart rate (bpm)	12-week LVEDV (ml)	12-week LVESV (ml)	12-week LVEF (%)	12-week Infarct size (%)	12-week Diastolic wall thickness (mm)	12-week Systolic wall thickness (mm)	12-week Wall motion (mm)
2515		104	44.68	28.7	35.77	15.1	5.2	6	5	119	55.88	37.06	33.69	14.9	4.9	4.6	2.7								
2545		130.7	43.4	30.48	29.76	14.2	4.5	4.5	0.2	99.2	55.78	33.7	39.59	10.5	4.9	4.2	0.5								
2548		153.1	34.89	21.01	39.78	16.3	5.3	5	1.3	115	61.87	37.86	38.81	14.5	4.4	4.4	0.7								
2666		50	35.22	22.48	36.17	15.1	4.1	5.5	2.2	69	33.29	20.97	37	13.5	4.4	4.9	1.9								
2668	Anemia	106	32.88	20.42	37.89	12.8	5.3	6	2.6	76.2	35.52	23.01	35.22	13.3	5.2	5.4	2.2								
2665	Anemia	102	30.59	21.02	31.29	14.5	5.9	6.1	2.6	80	46.77	30.41	34.99	11.6	5.9	6.5	1.7								
2585	Anemia	132.8	26.58	17.23	35.19	10	4.3	5.1	3.4	93.7	65.03	40.51	37.7	11.9	5.4	7.9	2.9								
2517		118.6	37.5	22.41	40.25	14.3	4.6	4.1	3.7	135	51.43	32.68	36.44	13.9	4.7	3.8	-0.4	81.2	57.8	37.56	35.02	15.9	6.4	5.6	0.3
2572		121	41.59	26	37.5	11.8	6	6.3	2.1	115.4	50.13	30.89	38.39	13.7	5	6	1.7	97	41.99	25.85	38.44	12.7	4.5	4.2	-0.1
2583		125	29.24	18.39	37.12	16.4	4.5	5.2	3	95.2	62.95	39.86	36.68	17.5	5.1	4.8	1.5	80.9	88.93	53.98	39.3	13.6	6.4	6.4	2.9
2469		115.3	27.13	16.35	39.76	12.7	5.7	6.7	3.6	111	31.04	16.51	46.82	12.5	5.3	6.8	2.4								
2520		109.2	44.35	26.12	41.11	12	5	6.1	3.1	100	52.51	28.18	46.34	12.3	6.7	7.5	2.5								
2584		148	47.44	28.45	40.02	12.8	4.9	6.3	3.8	102.7	64.46	38.59	40.13	9.1	5.6	5	4.5								
2578	lung infection	110.5	45.67	25.94	43.2	16.6	5.8	5.7	4.4	83.6	66.78	40.04	40.03	13.1	5.2	6.4	5.8								
2576	lung infection	120	46.33	26.86	42.01	7.1	6	6.7	5.1	78.4	76.5	48.91	36.07	9.1	5.7	7.1	6.4								
2498		124	31.96	19.35	39.45	14.3	Heart rate too fast			Heart rate too fast								85.1	65.47	37.98	41.98	8.2	5.8	6.7	5.3
2519		111	40.81	24.04	41.08	9	5.2	7.2	3.1	141.7	49.21	26.29	46.58	10.2	5.3	6.4	2.4	88	91.46	48.2	47.3	10.3	7	15.4	7.8
2577		75	44.16	25.64	41.93	11.2	5.9	8.2	1	113.1	41.27	22.23	46.14	11.7	6.1	6.7	-2	120.5	56.21	30.06	46.53	11.1	6.6	8.6	2.8
2573		138	37.29	21.73	41.72	11.2	4.9	5.7	2.7	76.8	51.64	30.47	41	7.2	5	7.2	3.6	74.3	72.91	41.29	43.37	7	6.4	7.1	4.1
2673		116	27.97	15.98	42.86	9.6	6.3	6.2	3.7	77	50.03	24.98	50.06	5.5	5.8	5.5	2.8	102	57.69	31.6	45.23	4.4	7.4		6

	Pre-MI					
	Heart rate (bpm)	LVEDV (ml)	LVESV (ml)	LVEF (%)	Diastolic wall thickness (mm)	Wall motion (mm)
1000	138.6	29.19	13.81	52.69	5.5	5
1001	127.9	34.79	15.63	55.07	4.8	4.6
1002	154.4	32.82	15.78	51.93	5	5.6
1003	175.4	20.65	9.38	54.61	4.8	4.5
1004	96.2	59.56	30.24	49.23	5	7.2
1005	111	42.85	20.94	51.14	4.8	5.6
1006	116.6	38.12	17.22	54.84	4.6	7
1007	112.6	49.6	23.45	52.73	4.9	6.3
1008	116.3	45.79	21.95	52.07	4.8	5
1009	75.2	56.63	27.58	51.29	4.2	5.1

Supplementary Table 4. Summary of in vivo electrophysiology measurements

	2667 (Sham)			2583 (media, 2 wks)			2585 (Media, 2 wks)			2515 (Media, 4 wks)		
	unipolar	bipolar	LLS	unipolar	bipolar	LLS	unipolar	bipolar	LLS	unipolar	bipolar	LLS
Apex	10	2.7	13.6	6.3	3.2	8	14.9	6	10.8	5	2.4	10.9
Midanterior	9.4	3.4	14.2	6.1	2	6.4	11.9	2.5	4.6	4.7	0.6	7.4
Anterobasal	12	3.8	13.4	6.9	4.2	5.8	12.5	3	-8.6	11.3	5	12.6
Midlateral	10.3	3.4	15.7	7.7	3.3	2.1	9.9	2.4	8.3	10.2	3.8	8.7
Basolateral	11.6	5.9	12.6	6.7	2.8	13.9	12.8	4.2	5.8	15.3	8.2	17.2
Midposterior	9.2	2.7	12.4	9.4	3.7	2.3	19.6	3.3	7.8	12.3	3.6	21.1
Posterobasal	14.4	3.8	8.6	9.1	3.7	24	9.4	2.8	10.4	9.3	4.3	16.7
Midseptal	12.9	1.8	12	12.4	2.8	8.8	13.9	4	13.6	13.3	3	13.5
Basoseptal	5.5	1.5	9.3	9.4	5	-0.5	4.6	1.8	7.9	8.4	2.1	8.7
Mean	10.59	3.22	12.42	8.22	3.41	7.87	12.17	3.33	6.73	9.98	3.67	12.98
	_			_			_			_		
Scar segments (bulleye) unipolar Voltage <5mV, bipV <0,5mV	0	0		0	0		1	0		2	0	
%scar unipV/ bipV (9 segments)	0.00	0.00		0.00	0.00		11.11	0.00		22.22	0.00	
	_			_			_			_		
Segments with normal movement (LLS>6%)	_		9	_		5	_		6	_		9
Hypokinetic segments (2-6% LLS)	_		0	_		3	_		2	_		0
Diskinetic segments(- %)	_		0	_		1	_		1	_		0
Akinetic segments (0-2% LLS)			0			0			0			0

	2573 (CCP, 2 wks)			2577 (CCP, 2 wks)			2584 (CCP, 2 wks)			2519 (CCP, 4 wks)			2519 (CCP, 10 wks)		
	unipolar	bipolar	LLS	unipolar	bipolar	LLS	unipolar	bipolar	LLS	unipolar	bipolar	LLS	unipolar	bipolar	LLS
Apex	21.6	6.4	7.6	13.6	4.8	2.1	10.2	4.3	9.9	7.6	4.1	12.7	7.3	5.4	18.8
Midanterior	12.5	3.4	1.8	14.7	3.7	8.9	7.1	2.3	6.6	9.7	5.7	9.6	13.5	3.6	20.2
Anterobasal	11.1	4.3	7.6	13.7	3.5	8.5	6.8	2.2	2.3	12.2	5.7	10.4	9.5	2.8	18.3
Midlateral	8.8	5	10.9	10.4	2.9	5.4	5.1	3.4	5.6	11.7	4.6	11.6	5.4	1.6	6.3
Basolateral	16.5	4.1	12.3	8.9	3.3	22.9	8.5	5.3	9.5	8.4	5.1	17.2	7.3	1.8	17.3
Midposterior	17.4	6.4	14.7	16.7	3.4	8.3	13.8	5.8	8.5	11.9	4.4	6.4	18.8	6.7	7.3
Posterobasal	10	3.1	14.8	9	3.6	9.8	6.8	4	11.6	11.2	5.2	11.1	11.6	3.7	16.8
Midseptal	14.3	5.5	11.4	14.9	5.7	-1.9	11.3	3.7	11.7	12.1	4.1	12.8	19.6	4	14.6
Basoseptal	15.4	4.1	7.7	11.5	3.2	11.4	6.1	2.8	5.3	7.9	3.7	9	12.6	4.1	9.4
Mean	14.18	4.70	9.87	12.60	3.79	8.38	8.41	3.76	7.89	10.30	4.73	11.20	11.73	3.74	14.33
	_			_			_			_			_		
Scar segments (bulleye) unipolar Voltage <5mV, bipV <0,5mV	0	0		0	0		0	0		0	0		0	0	
%scar unipV/ bipV (9 segments)	0.00	0.00		0.00	0.00		0.00	0.00		0.00	0.00		0.00	0.00	
	_			_			_			_			_		
Segments with normal movement (LLS>6%)	_		8	_		6	_		6	_		9	_		9
Hypokinetic segments (2-6% LLS)	_		0	_		2	_		3	_		0	_		0
Diskinetic segments(- %)	_		0	_		1	_		0	_		0	_		0
Akinetic segments (0-2% LLS)			1			0			0			0			0
