# Supplementary material for: Pluripotent stem cell-derived committed cardiac progenitors remuscularize damaged ischemic hearts and improve their function in pigs
Source: NPJ Regen Med. 2023 May 26;8:26. doi: 10.1038/s41536-023-00302-6 (PMC10219927; doi:10.1038/s41536-023-00302-6)
Supplement: Supplementary file 2 — nr-reporting-summary [file 41536_2023_302_MOESM2_ESM.pdf]

nature portfolio

Corresponding author(s): Lynn Yap and Karl Tryggvason

Last updated by author(s): Apr 28, 2023

Reporting Summary

Nature Portfolio wishes to improve the reproducibility of the work that we publish. This form provides structure for consistency and transparency in reporting. For further information on Nature Portfolio policies, see our Editorial Policies and the Editorial Policy Checklist.

Statistics

For all statistical analyses, confirm that the following items are present in the figure legend, table legend, main text, or Methods section.

n/a	Confirmed	
☐	☒	The exact sample size (*n*) for each experimental group/condition, given as a discrete number and unit of measurement
☐	☒	A statement on whether measurements were taken from distinct samples or whether the same sample was measured repeatedly
☐	☒	The statistical test(s) used AND whether they are one- or two-sided<br>*Only common tests should be described solely by name; describe more complex techniques in the Methods section.*
☐	☒	A description of all covariates tested
☐	☒	A description of any assumptions or corrections, such as tests of normality and adjustment for multiple comparisons
☐	☒	A full description of the statistical parameters including central tendency (e.g. means) or other basic estimates (e.g. regression coefficient) AND variation (e.g. standard deviation) or associated estimates of uncertainty (e.g. confidence intervals)
☐	☒	For null hypothesis testing, the test statistic (e.g. *F*, *t*, *r*) with confidence intervals, effect sizes, degrees of freedom and *P* value noted<br>*Give P values as exact values whenever suitable.*
☒	☐	For Bayesian analysis, information on the choice of priors and Markov chain Monte Carlo settings
☒	☐	For hierarchical and complex designs, identification of the appropriate level for tests and full reporting of outcomes
☒	☐	Estimates of effect sizes (e.g. Cohen's *d*, Pearson's *r*), indicating how they were calculated

Our web collection on statistics for biologists contains articles on many of the points above.

Software and code

Policy information about availability of computer code

Data collection	No software was used
Data analysis	STAR 2.5.2b, featureCounts, ComplexHeatmap R Bioconductor package (v2.6.2), CellRanger, Seurat V3 , ScaleData function, RunPCA , ChemoSpecMarkeR R package , FeaturePlot

For manuscripts utilizing custom algorithms or software that are central to the research but not yet described in published literature, software must be made available to editors and reviewers. We strongly encourage code deposition in a community repository (e.g. GitHub). See the Nature Portfolio guidelines for submitting code & software for further information.

Data

Policy information about availability of data

All manuscripts must include a data availability statement. This statement should provide the following information, where applicable:

- Accession codes, unique identifiers, or web links for publicly available datasets
- A description of any restrictions on data availability
- For clinical datasets or third party data, please ensure that the statement adheres to our policy

The bulk and single-cell RNA sequencing data is made available on Gene Expression Omnibus with accession number GSE204881.

Human research participants

Policy information about studies involving human research participants and Sex and Gender in Research.

Reporting on sex and gender	This information has not been collected
Population characteristics	See above
Recruitment	See above
Ethics oversight	See above

Note that full information on the approval of the study protocol must also be provided in the manuscript.

Field-specific reporting

Please select the one below that is the best fit for your research. If you are not sure, read the appropriate sections before making your selection.

☒ Life sciences ☐ Behavioural & social sciences ☐ Ecological, evolutionary & environmental sciences

For a reference copy of the document with all sections, see nature.com/documents/nr-reporting-summary-flat.pdf

Life sciences study design

All studies must disclose on these points even when the disclosure is negative.

Sample size	Sample size was calculated using animal sample size calculator (http://www.lasec.cuhk.edu.hk/sample-size-calculation.html) and also referred our previous publication (Ye et al. 2014, Cell Stem Cell)
Data exclusions	Left ventricular ejection fraction (LVEF) measurements of pigs that are under arrhythmia and MRI was unable to capture clear images were excluded. These parameters were predetermined.
Replication	We used various molecular techniques (western blots, bulk and single-cell RNAseq, qPCR) to confirm the CCP gene panels. Animal data (MRI and quantification of histology analysis) were determined by at least 2 independent individuals that were blinded to the treatment groups.
Randomization	Pigs were allocated into the CCP-transplanted or medium-control group by randomising the order of treatments and cardiac functional measurements into different surgery days. The animal surgeon, veterinarians, cardiac imaging specialists, electrophysiologists, and cardiac clinicians were unaware of the group allocation and treatment type.
Blinding	Investigators are blinded to the groups during data collection and/or analysis.

Reporting for specific materials, systems and methods

We require information from authors about some types of materials, experimental systems and methods used in many studies. Here, indicate whether each material, system or method listed is relevant to your study. If you are not sure if a list item applies to your research, read the appropriate section before selecting a response.

Materials & experimental systems

n/a	Involved in the study	
☐	☒	Antibodies
☐	☒	Eukaryotic cell lines
☒	☐	Palaeontology and archaeology
☐	☒	Animals and other organisms
☒	☐	Clinical data
☒	☐	Dual use research of concern

Methods

n/a	Involved in the study	
☒	☐	ChIP-seq
☒	☐	Flow cytometry
☐	☒	MRI-based neuroimaging

Antibodies

Antibodies used	Mouse monoclonal to POU5F1 (C-10) Santa Cruz Cat.#sc-5279; RRID:AB_628051<br>Mouse monoclonal to TRA-1-60 (Clone TRA-1-60) Millipore Cat.#MAB4360; RRID:AB_2119183<br>Mouse monoclonal to cardiac troponin T (clone 13-11) ThermoFisher Cat.#MS-295-p1; RRID:AB_61808

Rat monoclonal to ISL1 (NS1) DSHB Cat. #39.4D5, RRID:AB_2314683
Rabbit polyclonal to NKX2.5 (H-114) Santa Cruz Cat. # sc-14033, RRID:AB_650281
Mouse monoclonal to myosin DSHB Cat.#MF20; RRID: AB_2147781
Mouse monoclonal to a-Actinin (Clone EA-53) Sigma Cat.#A7811; RRID: AB_476766
Mouse monoclonal to connexin 43 BD Biosciences Cat.#610061; RRID:AB_397473
Rabbit monoclonal to Ku80 (clone EPR3468) Abcam Cat.#ab80592; RRID:AB_1603758
Rabbit polyclonal to cardiac troponin I (clone H-170) Santa Cruz Cat.#sc-15368; RRID:AB_793465
Rabbit polyclonal to Nkx-2.5 (clone H-114) Santa Cruz Cat.#sc-14033; RRID:AB_650281
Anti-GATA4 (1:1000) Abcam Cat. # ab124265, RRID:AB_11000793
Anti-ISL1 (1:1000) Abcam Cat. #86472, RRID:AB_1951287
Anti-NKX2.5 (1:250) Santa Cruz Cat. # sc-14033, RRID:AB_650281
Anti-MYH6 (1:1000) Abcam Cat. # ab50967, RRID:AB_942084
Anti-SLC8A1 (1:1000) Cell Signalling Cat. #55075-1-AP, RRID:AB_2881262
Anti-ACTN2 (1:1000) Sigma Cat. # A7811, RRID:AB_476766
Anti-ACTC1 (1:1000) Sigma Cat. # SAB5600071
Anti-ANKRD1 (1:250) Millipore Cat. # MABS1228
Anti-CRHBP (1:1000) Sigma Cat. # HPA046120, RRID:AB_10959760
Anti-TNNT2 (1:5000) Abcam Cat. # ab91605, RRID:AB_2050427
Anti-IGFBP7 (1:200) R&D Systems Cat. # AF1334, RRID:AB_2264436
Anti-CCDC80 (1:1000) R&D Systems Cat. # AF3410
Anti-TNNI1 (1:1000) Sigma Cat. # AV42117, RRID:AB_1858352
Anti-MYL4 (1:1000) Abcam Cat. # ab231800
Anti-actin (1:10000) Millipore Cat. #MAB1501R, RRID:AB_2223041
Anti-Ku80 conjugated 488 Abcam Cat. #ab198586
Anti-MLC2v Abcam Cat. #ab79935, RRID:AB_1952220
Anti-ACTN2 Sigma Cat. #A2172, RRID:AB_476695
Anti-N-cadherin Sigma Cat. #C3678, RRID:AB_258851
Anti-CX43 Sigma Cat. #6219C
Anti-CD31 Abcam Cat. #ab28364, RRID:AB_726362
Anti-TNNI3 Novus Cat. #NBP1-56641, RRID:AB_11035917
Anti-MLC2a Sigma Cat. #HPA013331, RRID:AB_1854245
Anti-Ki67 Abcam Cat. #ab15580, RRID:AB_443209
Anti-CD45 Bio-Rad Cat. #MCA1447, RRID:AB_2174248
Anti-CD20 Biocare Medical Cat. #ACR3004B
Anti-CD3 Dako Cat. # A0452, RRID:AB_2335677
Anti-PPH3 Cell Signaling Cat. #9701, RRID:AB_331535
Mouse IgG negative control Dako Cat.#X0944
Mouse IgM negative control Dako Cat.#X0942
Rabbit IgG negative control Biolegend Cat.#910801; RRID:AB_2722735
Goat anti-mouse IgG (H+L), AlexaFluor488 conjugated ThermoFisher Cat.#A11001, RRID: AB_2534069
Goat anti-mouse IgG (H+L), AlexaFluor647 conjugated ThermoFisher Cat.#A21235; RRID:AB_141693
Goat anti-rabbit IgG (H+L), AlexaFluor488 conjugated ThermoFisher Cat.#A11008; RRID:AB_143165
Goat anti-rabbit IgG (H+L), AlexaFluor647 conjugated ThermoFisher Cat.#A21244; RRID:AB_141663
Donkey anti-rabbit IgG (H+L), HRP conjugated ThermoFisher Cat.# SA1-200; RRID:AB_325994

Validation

These antibody is validated by the manufacture's website and our positive control samples.

Eukaryotic cell lines

Policy information about cell lines and Sex and Gender in Research

Cell line source(s)	H1 cells are bought from WiCell Institute
Authentication	Cell lines are not authenticated
Mycoplasma contamination	Cell lines were tested negative for mycoplasma contamination
Commonly misidentified lines (See ICLAC register)	No misidentified lines used in the study

Animals and other research organisms

Policy information about studies involving animals; ARRIVE guidelines recommended for reporting animal research, and Sex and Gender in Research

Laboratory animals	Either gender of 3-month-old Sus scrofa pigs weighing 13-15 kg
Wild animals	Study did not involve wild animals
Reporting on sex	Both female and male sus scrof pigs were used and randomly assigned to either CCP-transplanted or medium-control groups.
Field-collected samples	Study did not involve samples collected from the field.

March 2021

Ethics oversight | SingHealth's Institutional Animal Care and Use Committee (IACUC) (2018/SHS/1426)

Note that full information on the approval of the study protocol must also be provided in the manuscript.

Magnetic resonance imaging

Experimental design

Design type: MRI was performed to measure the left ventricular function of the pigs after myocardial infarction.

Design specifications: Cardiac Magnetic resonance imaging (MRI) was performed at 1, 4, and 12 weeks (for pigs with 12-week follow-up) post-surgery. Replicates number for week 1 (n=10 medium control, n=10 CCP transplanted), week 4 (n=10 medium control, n=10 CCP transplanted) and week 12 (n= 3 medium control, n=5 CCP transplanted) weeks post cell-transplantation

Behavioral performance measures: MRI imaging was performed by staff who were blinded to the animal groups.

Acquisition

Imaging type(s): Functional and perfusion

Field strength: 3 Tesla

Sequence & imaging parameters: Imaging was performed with a 3T Skyra MR Imaging System (Siemens Medical Solutions, Erlangen, Germany) scanner. The animal was placed head first, supine, with 18-channel body array coil around the chest. The CMR images were gated to the ECG and obtained during repeated breath-holds. The scans were performed at 1, 4 and 12-weeks. Late gadolinium enhancement) scans were done 8 mins after Dotarem injection

Area of acquisition: Whole heart

Diffusion MRI: ☐ Used ☒ Not used

Preprocessing

Preprocessing software: Global function (Left ventricular ejection fraction (LVEF)) was computed from the short-axis cine images by semi-automated segmentation of the LV endocardial and epicardial borders (from base to apex) at both end-diastole and end-systole using CVi42 analysis software (Circle Cardiovascular Imaging Inc., Canada) 10.

Normalization: No normalization was used because of the baseline scans and experimental pigs were different.

Normalization template: Data were not normalized

Noise and artifact removal: Physiological monitoring will be applied and recorded. Such as heart rate, sPO2, body temperature monitor by thermal catheter, heating fan, CO2%, IV access for 0.9% NaCl dripping and injecting contrast. If the heart rate is low, atropine (0.04mg/kg, i.v.) will be administered by the vets in NUS CM. The vital signs (HR, RR, ETC)2, ECG and temperature) will be continuously being monitored while the animal is anesthesied. The animal is also mechanically ventilated during the scans.

Volume censoring: Heart volume was not measured

Statistical modeling & inference

Model type and settings: n/a

Effect(s) tested: n/a

Specify type of analysis: ☐ Whole brain ☐ ROI-based ☐ Both

Statistic type for inference (See Eklund et al. 2016): n/a

Correction: n/a

Models & analysis

n/a	Involved in the study
☒	☐ Functional and/or effective connectivity
☒	☐ Graph analysis
☐	☒ Multivariate modeling or predictive analysis

Multivariate modeling and predictive analysis: Normalization PCA, dimensional reduction done using Seurat.
